# Squid Game Optimizer (SGO): a novel metaheuristic algorithm

**DOI:** 10.1038/s41598-023-32465-z

**Published:** 2023-04-01

**Authors:** Mahdi Azizi, Milad Baghalzadeh Shishehgarkhaneh, Mahla Basiri, Robert C. Moehler

**Affiliations:** 1 Department of Civil Engineering, University of Tabriz, Tabriz, Iran; 2 Department of Civil Engineering, Monash University, Clayton, VIC 3800 Australia; 3 Department of Civil Engineering, Near East University, Nicosia, Cyprus

**Keywords:** Mathematics and computing, Civil engineering

## Abstract

In this paper, Squid Game Optimizer (SGO) is proposed as a novel metaheuristic algorithm inspired by the primary rules of a traditional Korean game. Squid game is a multiplayer game with two primary objectives: attackers aim to complete their goal while teams try to eliminate each other, and it is usually played on large, open fields with no set guidelines for size and dimensions. The playfield for this game is often shaped like a squid and, according to historical context, appears to be around half the size of a standard basketball court. The mathematical model of this algorithm is developed based on a population of solution candidates with a random initialization process in the first stage. The solution candidates are divided into two groups of offensive and defensive players while the offensive player goes among the defensive players to start a fight which is modeled through a random movement toward the defensive players. By considering the winning states of the players of both sides which is calculated based on the objective function, the position updating process is conducted and the new position vectors are produced. To evaluate the effectiveness of the proposed SGO algorithm, 25 unconstrained mathematical test functions with 100 dimensions are used, alongside six other commonly used metaheuristics for comparison. 100 independent optimization runs are conducted for both SGO and the other algorithms with a pre-determined stopping condition to ensure statistical significance of the results. Statistical metrics such as mean, standard deviation, and mean of required objective function evaluations are calculated. To provide a more comprehensive analysis, four prominent statistical tests including the Kolmogorov–Smirnov, Mann–Whitney, and Kruskal–Wallis tests are used. Meanwhile, the ability of the suggested SGOA is assessed through the cutting-edge real-world problems on the newest CEC like CEC 2020, while the SGO demonstrate outstanding performance in dealing with these complex optimization problems. The overall assessment of the SGO indicates that the proposed algorithm can provide competitive and remarkable outcomes in both benchmark and real-world problems.

## Introduction

Real-world optimization problems are considered quite challenging tasks and intricate problems in almost every field, classifying them in miscellaneous categories, including constrained or unconstrained, single or multi-objective, continuous or discrete, and static or dynamic. In some of engineering and industrial applications, which can be formulated as optimization problems, researchers are attempting to optimize specific variables, whether to reduce costs and energy consumption or increase profit, production, efficiency, and performance. Consequently, it is imperative to have an efficient optimizer to guarantee that the best solutions are found. The central component of an optimizer is a search or optimization algorithm that is properly designed and executed to conduct the necessary exploration. For a long time, traditional search approaches have been used to solve optimization issues. Even though these strategies provide promising results in different real-world problems, they may face failure in more complicated optimization problems. Hence, to solve these complex problems, metaheuristic algorithms have been developed.

Generally, metaheuristic algorithms can be categorized into four primary groups. The first one is the evolutionary based metaheuristic algorithms, which simultaneously perform the search procedure using several initial points. Holland^1^ introduced the Genetic Algorithm (GA), which is a popular population-based metaheuristic algorithm inspired by Darwinian evolution theory. Differential Evolution (DE) algorithm is another well-known stochastic population-based algorithm for global optimization^2,3^. Furthermore, with the popularity of the Imperialist Competitive Algorithm^4^, there have been various more frequently used population-based algorithms, including Charged System Search^5–7^, Intelligent Water Drops^8^, Stochastic Paint Optimizer^9,10^, Political Optimizer^11^, Dynamic Virtual Bats Algorithm^12^, Ali Baba and the forty thieves^13^, Tiki-taka algorithm^14^, and Coronavirus Optimization Algorithm^15^. The second category is swarm-based metaheuristic algorithms, which are based on the social behaviour of diverse species in natural groups like ants, bees, birds, fishes, and termites. Particle Swarm Optimization (PSO)^16^ and Ant Colony Optimization (ACO)^17,18^ are prominent swarm-based algorithms While these algorithms imitate bird and ant colonies' aggregation and foraging behaviors, respectively^19^. Artificial Bee Colony (ABC)^20,21^, Al-Biruni Earth Radius^22^, Border Collie Optimization^23^, Stochastic Diffusion Search^24^, Glowworm Swarm Optimization^25^, Mountain Gazelle Optimizer^26^, Cuckoo Search^27^, Flower Pollination Algorithm^28^, and Black Widow Optimization Algorithm^29^ are other examples of this sort. Furthermore, physics-based metaheuristic algorithms are the third classification inspired by physics laws, such as heat transformation, gravitational force, particle motions, and wave propagation. Undoubtedly, the SA algorithm, inspired by annealing in solids’ analogy to the statistical mechanics, has gained much popularity in this category^30^. Big-Bang Big-Crunch algorithm, which is inspired by the theories of the evolution of the universe, is another example of a physics-based optimization algorithm^31^. Additionally, it is noteworthy to state that there are still various metaheuristic methods that take inspiration from the laws of physics that have been documented in literature. Some of the most common algorithms include Atomic Orbital Search^32–34^, Material Generation Algorithm^35–37^, Cyber-physical Systems^38^, Chaos Game Optimization^39,40^, Archimedes Optimization Algorithm^41^, Lichtenberg Algorithm^42^, Energy Valley Optimizer^43^, Crystal Structure Algorithm^44–46^, Thermal Exchange Optimization Algorithm^47^, Equilibrium Optimizer^48^, Weighted Vertices Optimizer^49^, and Lévy Flight Distribution^50^. Finally, the last group is human behavior-based metaheuristic algorithms. The development of certain human-based algorithms has been inspired by simulating various human behaviors, including Teaching–Learning-Based Optimization (TLBO)^51^, Cultural Algorithm (CA)^52^, and Harmony Search (HS)^53^. TLBO has been developed based on the teaching and learning activities between teachers and students. CA has been developed with an inspiration from human sociology. Similarly, HS has been developed by taking inspiration from the adjustments in tone made by musicians in a band. These algorithms aim to capture and replicate the effective aspects of human behavior in problem-solving and optimization tasks. By simulating these human behaviors, they provide new and effective approaches for solving complex problems in various fields.

The main contribution of this paper is to propose a novel search method for optimization purposes in which an intelligent procedure is conducted for finding the best optimal values of different optimization problems. The applicability of the proposed method in dealing with difficult optimum design problems is another key contribution of this paper so the capability of the novel algorithm will be assessed through different procedures. In this research, a novel population-based metaheuristic optimization algorithm, called Squid Game Optimizer (SGO), is proposed. The proposed algorithm is inspired by the strategy of the squid game. It finds the optimal solution from a population of candidate solutions; each solution is referred to as an offensive individual attempting to overcome the defensive population. As a result, an offensive player with a higher value for the objective function could beat others. Finally, the best offensive player will be the winner at the end of the iteration. This concept is being used to develop a metaheuristic algorithm for the first time. This study introduces a new and inspiring research approach by assessing the complexity level of test functions, which has not been previously attempted. Evaluating the performance of numerous algorithms under different scenarios is a challenging task, as it is not possible to confirm the superiority of any algorithm with complete certainty. To address this, the study employs 25 unconstrained mathematical test functions, each with 100 dimensions, to evaluate the proposed algorithm's effectiveness. To ensure the statistical validity of the results, this study conducts 100 independent optimization runs and computes measures such as the mean, standard deviation, and required objective function evaluations. A predefined stopping criterion is utilized, taking into account both the maximum number of objective function evaluations and the tolerance for the global best values of the problems being considered. Finally, a number of well-known statistical analyses such as the Kolmogorov–Smirnov, Wilcoxon, Mann–Whitney, and Kruskal–Wallis tests are employed for comparative purposes. In light of the inadequacy of evaluation test functions in newly developed metaheuristic algorithms, this study seeks to address this shortcoming by assessing the complexity level of two recent Competitions on Evolutionary Computation (CEC). Specifically, the CEC 2020 competition on bound constraint optimization^54^ is used to assess the complexity of the SGO, while the CEC 2020 competition on real-world optimization^55^ is used to compare the performance of the SGO against that of cutting-edge algorithms. By leveraging these competitions, the study aims to improve the evaluation of metaheuristic algorithms and enhance their effectiveness in solving real-world optimization problems.

It should be noted that the metaheuristic algorithms are some sorts of stochastic methods in which randomization procedures are the key aspect of these approaches while multiple random numbers are generated through the optimization procedure in order to reach the global optimal point. For this purpose, the main limitation of these algorithms including the SGO is the reliability of them in initial random solutions created by these algorithms. The initial solutions near to global optimal can make the algorithms converge to better solutions while the solutions far from the global optima can underestimate the capability of the algorithm. Hence, the statistical procedures by means of multiple optimization runs can overcome this drawback so the SGO is justified through adequate statistical analysis.

The main contribution of this paper is the proposition of a novel metaheuristic algorithm in which the strategies and rules of the Squid Game as one of the Korean ancient games are used as the inspirational concept. The applicability of this algorithm in dealing with different kinds of optimization problems is the main challenge of this population-based algorithm in which the solution candidates are divided into two groups of offensive and defensive players while the offensive player goes among the defensive players to start a fight which is modeled through a random movement toward the defensive players. The results of this algorithm in dealing with mathematical problems with 100 dimensions demonstrates the capability of this algorithm in dealing with large search domains while solving the engineering design problems by this algorithm can guarantee the possibility of using this algorithm in real-world applications which is a main concern of the researchers based on the recent challenges in developing intelligent techniques for engineering optimization purposes.

The rest of the paper is structured in the following manner. Section "Related works" and "Squid Game Optimizer Algorithm" describe the related works and SGO developed in this study, respectively. Mathematical test problems and statistical outcomes are provided and discussed in Sections "Mathematical test functions" and "Numerical results of the mathematical functions". The complete statistical analysis is given in Section "Statistical Analysis", while the complexity analysis is conducted in Section "Computational complexity and cost analysis". The real-world problems of CEC are demonstrated in Section "Real-world constrained optimization problems of CEC 2020" while Sect.  9 summaries this study's primary outcomes and propose future research directions.

## Related works

Nowadays, metaheuristic algorithms have been utilized for the optimization of intricate real-world scientific and engineering problems; since they do not need complicated mathematical expressions, they can deal with constraints much more simply than traditional methods, and they all try to favour the search for the global optimum solution rather than local ones. In health and medicine, Canayaz^56^ used particle swarm and gray wolf optimization algorithms to discover Covid-19 at an early stage.; Basu et al.^57^ employed Harris Hawks Optimization algorithm for identifying COVID-19 from radiological images; Bandyopadhyay et al.^58^ employed a hybrid approach of this algorithm and Simulated Annealing algorithm to detect COVID-19 from CT scan images; and Hosseini et al.^59^ used COVID-19 optimizer Algorithm for reducing the number of COVID-19-infected regions and thus slowing the spread of the disease. Moreover, metaheuristic algorithms have been considered for solving different problems in medicine, including healthcare systems^60^, medical image segmentation^61–63^, optimization of cognitive big data healthcare^64^, diagnosis of brain tumors and breast cancer^65,66^, diagnosis of Parkinson's disease^67^, and Brain Cine-MRI^68^. Nonetheless, in chemistry, Chen et al.^69^ used ranking-based differential evolution algorithms for dynamic optimization problems; Cheema et al.^70^ employed a GA for optimization of glucose to gluconic acid fermentation; Mohd Zain et al.^71^ used Backtracking Search Algorithm for optimization of fed-batch fermentation processes; Geem and Kim^72^ used HS algorithm for wastewater treatment optimization for fish migration; and Piotrowski et al.^73^ applied the ABC algorithm and the Direct Search Algorithm to optimize the biological procedures in wastewater sequencing batch reactors.

Similarly, researchers have used metaheuristic algorithms to solve and optimize a plethora of intricate engineering problems in recent years. In civil engineering, Çerçevik et al.^74^ used three different algorithms, including Cuckoo Search Algorithm, Whale Optimization Algorithm (WOA), and Grey Wolf Optimizer to optimize the parameters of seismic isolated structures. Kaveh and Mahdavi^75^ optimized arch dams’ shape under earthquake loading with the Charged System Search and PSO algorithms. Azizi et al.^40^ conducted truss structures’ shape and size optimization employing the Chaos Game Optimization approach. Azizi et al.^76^ optimized the design of engineering problems using the Atomic Orbital Search algorithm. Kaveh and Khosravian^77^ used Vibrating Particles System algorithm to optimize truss structures’ layout and size. Gandomi et al.^78^ utilized the Cuckoo Search algorithm for five truss design optimization problems. Furthermore, Zhang et al.^79^ utilized the PSO algorithm to optimise concrete mixture proportions. Sun et al.^80^ applied the ABC algorithm to anticipate and optimise the concrete samples’ compressive strength. Chou et al.^81^ prognosticated the shear strength in reinforced concrete deep beams. In mechanical engineering, Muthu et al.^82^ conducted an optimal tolerance design for assembly with the goal of minimizing quality loss and manufacturing cost, using both GA and PSO algorithms. Hassan et al.^83^ applied the ACO algorithm to optimise pressure vessels' optimum design. Acharya et al.^84^ optimized proportional–integral–derivative control scheme parameters to regulate the DC motor’s speed. Pham et al.^85^ used different metaheuristic optimization algorithms in the functionally graded sandwich porous beams’ optimum design. Furthermore, other application of metaheuristic algorithms could be found in Refs.^86–114^ including the Firefly Algorithm (FA).

The development of a new metaheuristic approach is crucial because it can efficiently and effectively explore the search space. A smart search process is needed to comprehensively evaluate all the search areas, as well as to branch out into previously unexplored regions with high-quality solutions. These two processes are known as intensification and diversification, respectively. Diversification refers to exploring regions that have not been adequately explored, while intensification involves utilizing the collected information by the metaheuristic at a particular point in time^115^. The exploration phase of a metaheuristic algorithm involves a systematic search for promising regions of the search space. During the exploration phase, the algorithm generates a diverse set of candidate solutions by using various strategies such as mutation, crossover, and local search. These techniques allow the algorithm to explore the search space and move towards promising regions that are likely to contain high-quality solutions. The exploration stage of a metaheuristic algorithm holds paramount importance as it plays a crucial role in preventing the algorithm from getting stuck in local optima and elevating the quality of solutions. By traversing diverse regions of the search space, the algorithm can discover superior solutions that are closer to the global optimum. Following the exploration phase, the algorithm usually transitions to an exploitation phase, where it focuses its search on the most favorable regions of the search space The balance between exploration and exploitation is an essential factor that determines the effectiveness of the algorithm in finding near-optimal solutions^116,117^. A proper balance of these two phases may guarantee that the global optimum is reached. Even though there are many metaheuristic algorithms, new algorithms are constantly necessary. As highlighted in No Free Lunch theory states, there remains no specific technique for obtaining the optimal answer for practically all optimization problems; developing new metaheuristic optimization algorithms remains an ongoing subject^118,119^.

## Squid Game Optimizer Algorithm

### Inspiration

Squid game, also known as *ojingeo*, is based on a Korean children's playground game and is basically a blend of tag and hopscotch. It is a multiplayer game with two primary goals: either for the attackers to complete the attack's goal or for the teams to eliminate each other. Squid game is often played on large and sandy fields, although players can play in vast, open, indoor, or outdoor areas. Meanwhile, there remain no recommendations or guidelines regarding the size and dimensions of the playground. The squid-shaped playfield seems to be approximately half the size of a typical basketball court, based on the history of the squid game.

**Figure 1:**
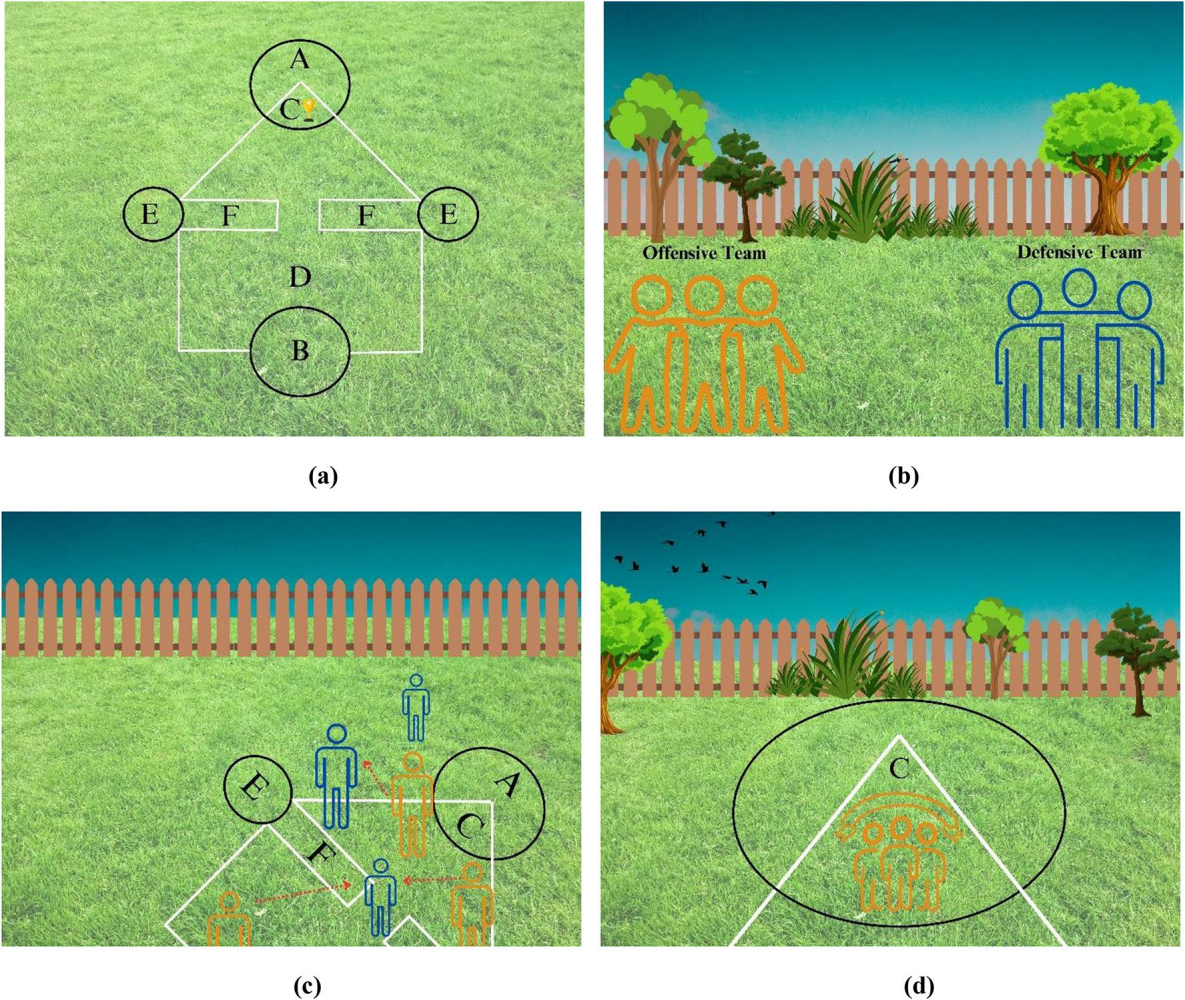
The schematic presentation of Squid Game.

As shown in Fig. 1a, squid game court resembles its namesake with a large body and tapering head. Begin by drawing the squid's head, which has a triangular tip and the square base of its body, indicated as section D. Section A is the big, rounded element, whereas section C is the triangle point within the circle. Section B is laid out at the exact centre of the horizontal line along the bottom of the squid, and it should be spacious enough for many participants to stand there, which is the main door or entry gate to the squid game court. Nevertheless, the bridge, which players may access via section E, is the exception to this rule. First and foremost, all players are divided into two groups: offensives and defensives (Fig. 1b). The offensive player must enter into section B as an offensive player while avoiding the other defensive players. The offensive team wins when any offensive player gets in section C with both feet, and to formally win the game, the offensive player must shout "Hurray." Defensive players can do whatever to get the offensive players off the field, whether it is pushing or pulling them over a line or knocking them down (Fig. 1c). If the defensive player can clear out all offensive players before reaching section C (Fig. 1d), they win. Only the defensive and offensive feet are permitted to contact the ground throughout the squid game. Players that are pushed, pulled, or tripped to the ground are ejected from the game.

This game begins by standing in section A. However, offensive players cannot bunny hop into section C and win the game immediately. They must exit section A and go to the other end of the court, where they may enter via section B. Subsequently, the offensive player in section D hop on 1 foot outside the court, making the game more equitable for the defensive team and equalizing the playing field for both teams. An offensive player could jump over the whole area F to play with both feet, which these parts are also called the bridge. Any offensive player who successfully leaps over this bridge on one foot is exempt from the 1-foot rule and may move on both feet for the remainder of the game. Offensive players may shove or force defensive players down if they get the chance, and reaching section C will be simpler if defensive players are removed.

Defensive players are allowed to move freely on both feet within the majority of the play area, which includes the circular sections. However, this defensive advantage comes to an end when any player exits sections D, A, B, or E. Similar to offensive players, defensive players must hop on one foot outside the court, and their team wins when they successfully eliminate all the offensive players.

### Mathematical model

The mathematical presentation of the SGO as an optimization algorithm is explained in detail in this part using the strategy of Squid Game which has been discussed in detail in the earlier section. In the first stage, the initialization procedure is carried out as follows while the search space is considered as a particular part of the playground and the solution candidates (X_i_) are assumed to be players:

1 $$X=\left[\begin{array}{c}{X}_{1}\\ {X}_{2}\\ \vdots \\ {X}_{i}\\ \vdots \\ {X}_{n}\end{array}\right]=\left[\begin{array}{c}{x}_{1}^{1} {x}_{1}^{2} \cdots {x}_{1}^{j} \cdots {x}_{1}^{d}\\ {x}_{2}^{1} {x}_{2}^{2} \cdots {x}_{2}^{j} \cdots {x}_{2}^{d}\\ \vdots \vdots \vdots \ddots \vdots \\ {x}_{i}^{1} {x}_{i}^{2} \cdots {x}_{i}^{j} \cdots {x}_{i}^{d}\\ \vdots \vdots \vdots \ddots \vdots \\ {x}_{n}^{1} {x}_{n}^{2} \cdots {x}_{n}^{j} \cdots {x}_{n}^{d}\end{array}\right], \left\{\begin{array}{c}\begin{array}{c}i=\mathrm{1,2},\dots ,n.\end{array}\\ j=\mathrm{1,2},\dots ,d.\end{array}\right.$$

2 $${x}_{i}^{j}={x}_{i,min}^{j}+rand.\left({x}_{i,max}^{j}-{x}_{i,min}^{j}\right), \left\{\begin{array}{c}\begin{array}{c}i=\mathrm{1,2},\dots ,n.\end{array}\\ j=\mathrm{1,2},\dots ,d.\end{array}\right.$$

where $n$ indicates players’ total number (solution candidates) in the playground (search space); $d$ is the considered problem’s dimension; ${x}_{i}^{j}$ refers to the *jth* decision variable used to determine the *ith* candidate’ initial position;${x}_{i,max}^{j}$ and ${x}_{i,min}^{j}$ are the upper and lower bounds of the *jth* variable in the *ith* candidate; $rand$ refers to a random number that is uniformly distributed within the range of [0, 1].

During the second stage of the algorithm, the players are separated into two equally sized groups: Offensives (Off) and Defensives (Def). The mathematical representation of these components is provided below:

3 $${X}^{Off}=\left[\begin{array}{c}{X}_{1}^{Off}\\ {X}_{2}^{Off}\\ \vdots \\ {X}_{i}^{Off}\\ \vdots \\ {X}_{m}^{Off}\end{array}\right]=\left[\begin{array}{c}{x}_{1}^{1} {x}_{1}^{2} \cdots {x}_{1}^{j} \cdots {x}_{1}^{d}\\ {x}_{2}^{1} {x}_{2}^{2} \cdots {x}_{2}^{j} \cdots {x}_{2}^{d}\\ \vdots \vdots \vdots \ddots \vdots \\ {x}_{i}^{1} {x}_{i}^{2} \cdots {x}_{i}^{j} \cdots {x}_{i}^{d}\\ \vdots \vdots \vdots \ddots \vdots \\ {x}_{m}^{1} {x}_{m}^{2} \cdots {x}_{m}^{j} \cdots {x}_{m}^{d}\end{array}\right], \left\{\begin{array}{c}\begin{array}{c}i=\mathrm{1,2},\dots ,m.\end{array}\\ j=\mathrm{1,2},\dots ,d.\end{array}\right.$$

4 $${X}^{Def}=\left[\begin{array}{c}{X}_{1}^{Def}\\ {X}_{2}^{Def}\\ \vdots \\ {X}_{i}^{Def}\\ \vdots \\ {X}_{m}^{Def}\end{array}\right]=\left[\begin{array}{c}{x}_{1}^{1} {x}_{1}^{2} \cdots {x}_{1}^{j} \cdots {x}_{1}^{d}\\ {x}_{2}^{1} {x}_{2}^{2} \cdots {x}_{2}^{j} \cdots {x}_{2}^{d}\\ \vdots \vdots \vdots \ddots \vdots \\ {x}_{i}^{1} {x}_{i}^{2} \cdots {x}_{i}^{j} \cdots {x}_{i}^{d}\\ \vdots \vdots \vdots \ddots \vdots \\ {x}_{m}^{1} {x}_{m}^{2} \cdots {x}_{m}^{j} \cdots {x}_{m}^{d}\end{array}\right], \left\{\begin{array}{c}\begin{array}{c}i=\mathrm{1,2},\dots ,m.\end{array}\\ j=\mathrm{1,2},\dots ,d.\end{array}\right.$$

where $m$ is players’ total number in each group in the game; ${X}_{i}^{Off}$ is the *ith* offensive player; ${X}_{i}^{Def}$ is the *ith* defensive player.

Subsequently, after commencing the game, one offensive player goes among the defensive players to start a fight. It is worthwhile to mention that each offensive player has to move and fight with a single foot while the defensives are free to play with both feet. The mathematical representation of these components is provided below:

5 $$DG=\frac{\sum_{i=1}^{m}{X}_{i}^{Def}}{m}, i=\mathrm{1,2},\dots ,m$$

6 $${X}_{i}^{OffNew1}=\frac{{X}_{i}^{Off}+{r}_{1}\times DG-{r}_{2}\times {X}_{{r}_{3}}^{Def}}{2}, i=\mathrm{1,2},\dots ,m$$

where $DG$ is the Defensive Group which mimics the crowd of defensive players; ${X}_{i}^{OffNew}$ is the upcoming *ith* offensive player’ position vector (${X}_{i}^{Off}$) in the playground;${\mathrm{r}}_{1}$ and ${\mathrm{r}}_{2}$ are two random numbers within the bounds of [0, 1] which represents the capability of the offensive players in reaching any position between the $DG$ and a randomly selected defensive player (${X}_{{r}_{3}}^{Def}$); r_3_ is a random integer number ranging from 1 to $m$.

**Figure 2:**
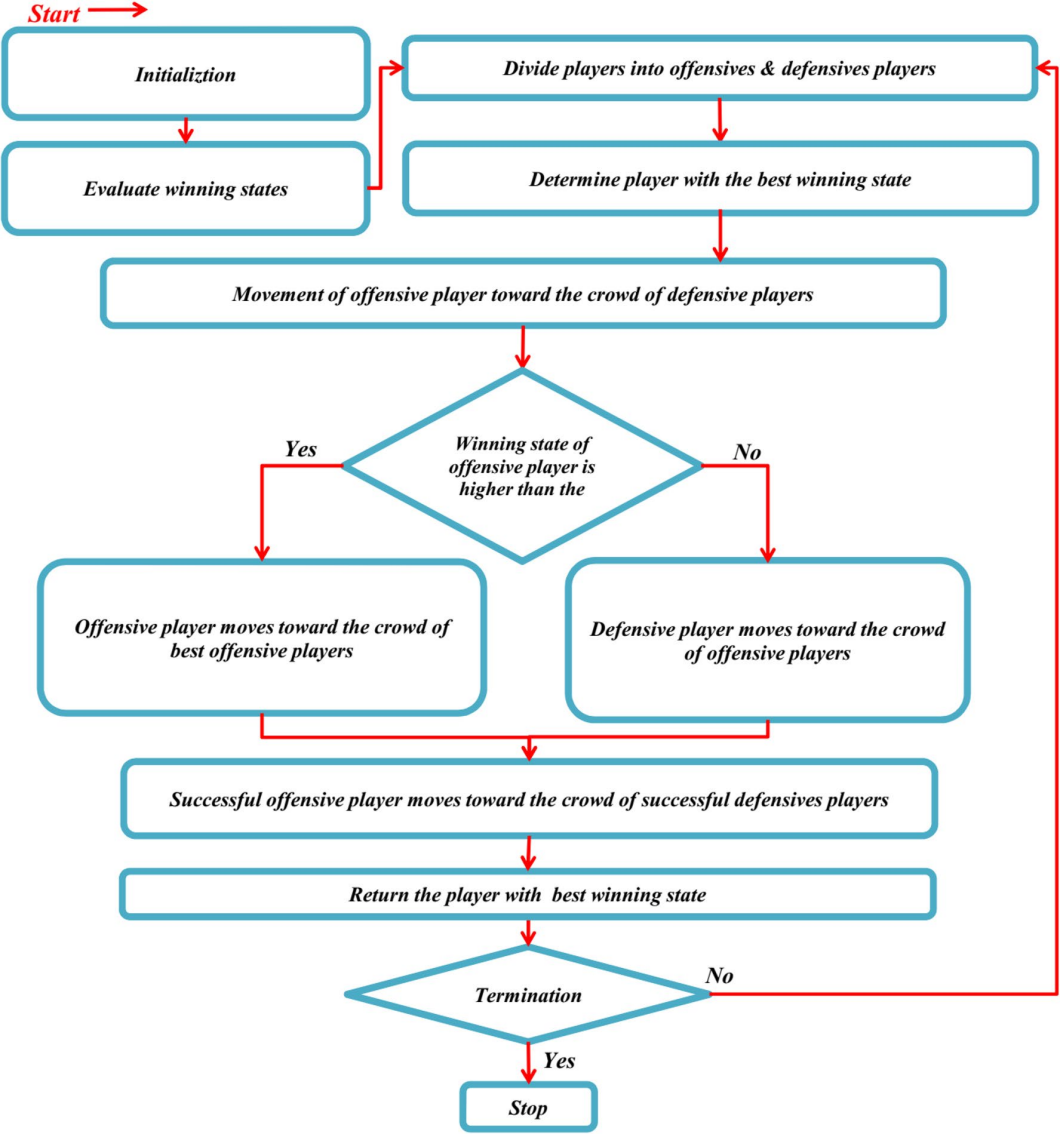
Flowchart of the SGO.

In the next step, after fighting between the *ith* offensive player (${X}_{i}^{Off}$) and a specific defensive player (${X}_{{r}_{3}}^{Def}$), the objective function evaluation for each player is carried out and identified as the Winning State (WS) of the players. If the wining state of the defensive player is lower than the winning state of the offensive player (${WS}_{i}^{Def}\le {WS}_{i}^{Off}$), the offensive player is assumed as the winner of the game and join the Successful Offensive Group ($SOG$) (point C in Fig. 2) regarding the primary rules of the squid game while the offensive player can use both feet for this purpose. The mathematical representation of these facets can be expressed as:

7 $${X}^{SccOff}=\left[\begin{array}{c}{X}_{1}^{SccOff}\\ {X}_{2}^{SccOff}\\ \vdots \\ {X}_{i}^{SccOff}\\ \vdots \\ {X}_{o}^{SccOff}\end{array}\right]=\left[\begin{array}{c}{x}_{1}^{1} {x}_{1}^{2} \cdots {x}_{1}^{j} \cdots {x}_{1}^{d}\\ {x}_{2}^{1} {x}_{2}^{2} \cdots {x}_{2}^{j} \cdots {x}_{2}^{d}\\ \vdots \vdots \vdots \ddots \vdots \\ {x}_{i}^{1} {x}_{i}^{2} \cdots {x}_{i}^{j} \cdots {x}_{i}^{d}\\ \vdots \vdots \vdots \ddots \vdots \\ {x}_{o}^{1} {x}_{o}^{2} \cdots {x}_{o}^{j} \cdots {x}_{o}^{d}\end{array}\right], \left\{\begin{array}{c}\begin{array}{c}i=\mathrm{1,2},\dots ,o.\end{array}\\ j=\mathrm{1,2},\dots ,d.\end{array}\right.$$

8 $$SOG=\frac{\sum_{i=1}^{o}{X}_{i}^{SccOff}}{o}, i=\mathrm{1,2},\dots ,o$$

9 $${X}_{i}^{OffNew2}={X}_{i}^{OffNew1}+{r}_{1}\times SOG-{r}_{2}\times BS i=\mathrm{1,2},\dots ,m$$

where $o$ is the number of successful offensive players in $SOG$ which mimics the crowd of successful offensive players; ${X}_{i}^{OffNew2}$ shows the upcoming position vector of the *ith* offensive player (${X}_{i}^{OffNew1}$); $BS$ indicates the best solution candidate or the most successful offensive player in the $SOG$; ${\mathrm{r}}_{1}$ and ${\mathrm{r}}_{2}$ are two random numbers in the range of [0, 1].

If the winning state of the defensive player is higher than the winning state of the offensive player (${WS}_{i}^{Def}>{WS}_{i}^{Off}$), the defensive player is considered as the winner of the game and join the Successful Defensive Group ($SDG$). The defensive players in this group are assumed to protect the critical point of the playground called the bridge (Point F in Fig. 2). Meanwhile, the successful defensive players go into the crowd of offensive players to be ready for starting a new fight. The mathematical presentation of these aspects are as follows:

10 $$SDG=\left[\begin{array}{c}{X}_{1}^{SccDef}\\ {X}_{2}^{SccDef}\\ \vdots \\ {X}_{i}^{SccDef}\\ \vdots \\ {X}_{p}^{SccDef}\end{array}\right]=\left[\begin{array}{c}{x}_{1}^{1} {x}_{1}^{2} \cdots {x}_{1}^{j} \cdots {x}_{1}^{d}\\ {x}_{2}^{1} {x}_{2}^{2} \cdots {x}_{2}^{j} \cdots {x}_{2}^{d}\\ \vdots \vdots \vdots \ddots \vdots \\ {x}_{i}^{1} {x}_{i}^{2} \cdots {x}_{i}^{j} \cdots {x}_{i}^{d}\\ \vdots \vdots \vdots \ddots \vdots \\ {x}_{p}^{1} {x}_{p}^{2} \cdots {x}_{p}^{j} \cdots {x}_{p}^{d}\end{array}\right], \left\{\begin{array}{c}\begin{array}{c}i=\mathrm{1,2},\dots ,p.\end{array}\\ j=\mathrm{1,2},\dots ,d.\end{array}\right.$$

11 $$OG=\frac{\sum_{i=1}^{m}{X}_{i}^{Off}}{m}, i=\mathrm{1,2},\dots ,m)$$

12 $${X}_{i}^{DefNew1}={X}_{i}^{Def}+{r}_{1}\times OG-{r}_{2}\times {X}_{{r}_{3}}^{Off} i=\mathrm{1,2},\dots ,m$$

where $OG$ is the Offensive Group which mimics the crowd of offensive players; ${X}_{i}^{DefNew1}$ is the upcoming *ith* defensive player’ position vector (${X}_{i}^{Def}$) in the playground (search space);${\mathrm{r}}_{1}$ and ${\mathrm{r}}_{2}$ are two random numbers in the range of [0, 1] which represents the capability of the defensive players in reaching any position between the $OG$ and a randomly selected offensive player (${X}_{{r}_{3}}^{Off}$) to participate in a new fight; r_3_ is a random integer number ranging from 1 to $m$.

To intelligently tune the exploration and exploitation phased of the proposed algorithm, another searching loop is implemented in the algorithm in which the offensive players in $SOG$ try to pass the bridge which is protected by the defensive players in $SDG$. For this purpose, a position updating procedure is carried out for all of the offensive players in $SOG$ by moving toward one specific defensive player in $SDG$ (which mimics the bridge passing process) and the best so far found solution candidate (which mimics the reward for the offensive player to pass the bridge). The mathematical presentation of these aspects are as follows:

13 $${X}_{i}^{OffNew3}={X}_{i}^{SccOff}+{r}_{1}\times BS-{r}_{2}{\times X}_{k}^{SccDef} \left\{\begin{array}{c}\begin{array}{c}i=\mathrm{1,2},\dots ,o.\end{array}\\ k=\mathrm{1,2},\dots ,p.\end{array}\right.$$

where $o$ and $p$ is the number of successful offensive and deffensive players in $SOG$ and $SDG$ respectively; ${X}_{i}^{OffNew3}$ shows the upcoming position vector of the *ith* successful offensive player (${X}_{i}^{OffNew1}$) which passes the bridge; $BS$ indicates the best solution candidate or the most successful offensive player in the $SOG$; ${\mathrm{r}}_{1}$ and ${\mathrm{r}}_{2}$ are two random numbers in the range of [0, 1].

**Figure 3:**
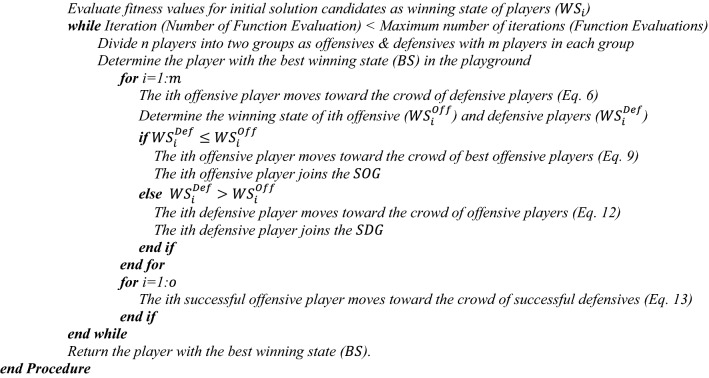
Pseudo code of the SGO.

In order to handle the situation where the solution variables (${\mathrm{x}}_{\mathrm{i}}^{\mathrm{j}}$) do not satisfy the boundary conditions, a mathematical flag has been formulated. This flag directs the adjustment of the boundary for those variables which violate the range of variables. Besides, the termination criteria can be determined as a predefined value for the number of iterations or the number of function evaluations after which the optimization process is ended. The SGO method is described in detail below, while the flowchart and pseudo-code are shown in Figs. 2 and 3.

- Step 1: The initial positions of potential solutions, denoted as (X_i_) or the "players" in the search space, are established through a random selection process.
- Step 2: The players are divided into two groups with equal populations, namely offensives (Off) and defensives (Def).
- Step 3: Each offensive player moves toward the crowd of a defensive player and one specific defensive player in order to start a fight.
- Step 4: The objective function for each offensive and defensive player are evaluated and indicated as the Winning State (WS).
- Step 5: The offensive players are able to join the Successful Offensive Group ($SOG$) if their WS is higher than that of the defensive player and moves toward the best offensive players in the playground.
- Step 6: The defensive players can join the Successful Defensive Group (*SDG*) if their WS is higher than that of the offensive player and moves toward the crowd of offensive players and one specific offensive player to be ready for another fight.
- Step 7: For each successful offensive player in the $SOG$, its position updating procedure is conducted by moving into the crowd of successful defensive players to pass the bridge.
- Step 8: The terminating criterion is checked.

## Mathematical test functions

To thoroughly examine the SGO algorithm, 25 unconstrained mathematical test functions, commonly used in global optimization, were selected, each with 100 dimensions. Table 1 presents a brief overview of these functions, and their full mathematical expressions have been documented by Jamil and Yang^120^, Jamil et al.^121^, Yang^122^, Liang et al.^123^, Talatahari and Azizi^39^, and Talatahari et al.^44^.

**Table 1:** The mathematical test functions used in this study possess certain fundamental characteristics.

No	Name	Type	R	Min
*F*_*1*_	Ackley 1	D, NS, C, Sc, M	[− 35, 35]	0
*F*_*2*_	Alpine 1	ND, S, NSc, U, C	[− 10, 10]	0
*F*_*3*_	Brown	NS, Sc, D, C, U	[− 1, 4]	0
*F*_*4*_	Chung Reynolds	D, PS, Sc, C, U	[− 100, 100]	0
*F*_*5*_	Csendes	M, Sc, S, D, C	[− 1, 1]	0
*F*_*6*_	Deb 1	S, D, C, Sc, M	[− 1, 1]	− 1
*F*_*7*_	Dixon & Price	NS, Sc, C, D, U	[− 10, 10]	0
*F*_*8*_	Extended Easom	M, NSc, C, D, S	[− 2π, 2π]	− 1
*F*_*9*_	Exponential	M, NS, Sc, C, D	[− 1, 1]	− 1
*F*_*10*_	Griewank	M, Sc, NS, D, C	[− 100,100]	0
*F*_*11*_	Holzman 2	S	[− 10, 10]	0
*F*_*12*_	Hyper-ellipsoid	U, C	[− 500, 500]	0
*F*_*13*_	Inverted cosine wave	NS	[− 10, 10]	− 99
*F*_*14*_	Levy 8	NS	[− 10, 10]	0
*F*_*15*_	Mishra 1	M, Sc, NS, C, D	[0, 1]	2
*F*_*16*_	Pathological	M, NSc, NS, C, D	[− 100, 100]	0
*F*_*17*_	Pint´er	M, Sc, NS, C, D	[− 10, 10]	0
*F*_*18*_	Powell Singular	U, Sc, NS, C, D	[− 4, 5]	0
*F*_*19*_	Powell Sum	U, Sc, S, C, D	[− 1, 1]	0
*F*_*20*_	Rastrigin	M, S, D, C	[− 5.12, 5.12]	0
*F*_*21*_	Qing	M, C, D, Sc, S	[− 500, 500]	0
*F*_*22*_	Quintic	M, NSc, S, C, D	[− 10, 10]	0
*F*_*23*_	Rosenbrock	U, C, D, NS, Sc	[− 30, 30]	0
*F*_*24*_	Salomon	NS, Sc, M, C, D	[− 100, 100]	0
*F*_*25*_	Schumer Steiglitz	U, S, Sc, C, D	[− 100, 100]	0

A total of 16 of the newest and most significant metaheuristic algorithms in optimization are used to assess the SGOA's overall performance compared to other metaheuristics, including the ABC^124^, ACO^17^, FA, GA^1^, PSO^125^, and WOA algorithms^126^. It is also worth mentioning that parameter tuning is imperative for some of these algorithms to have reasonable performance, extracting these parameters from the literature. Table 2 indicates the parameter presentation of the mentioned algorithms.

**Table 2:** Parameter presentation of the alternative metaheuristic algorithms.

Algorithms	Parameter	Definition	Value
*FA*	${N}_{pop}$	Number of fireflies	50
	$\gamma$	Light absorption coefficient	1
	$\beta$	Attraction coefficient base value	2
	$\alpha$	Mutation coefficient	0.2
	${\alpha }_{damp}$	Mutation coefficient damping ratio	0.98
	$\delta$	Uniform mutation range	± 0.05
*GA*	${N}_{pop}$	Number of population	50
	${p}_{c}$	Crossover percentage	0.8
	${p}_{m}$	Mutation percentage	0.3
	$\mu$	Mutation rate	0.02
	$\beta$	Roulette wheel selection pressure	1
*ABC*	${N}_{pop}$	Colony size	50
	$\alpha$	Acceleration coefficient upper bound	1
*ACO*	${N}_{Ant}$	Number of ants	50
	$\alpha$	Pheromone exponential weight	1
	$\beta$	Heuristic exponential weight	1
	$\rho$	Evaporation rate	0.05
*PSO*	*NB*	Swarm size	50
	*W*	Initial inertia	0.5
	*C*_*1*_	Cognitive coefficient	1.5
	*C*_*2*_	Social coefficient	1.5
*WOA*	${N}_{pop}$	Number of search agents	50

## Numerical results of the mathematical functions

In the current section, we present the outcomes of the SGO algorithm and six other metaheuristic algorithms on the 25 mathematical test functions that were investigated. The experiments were carried out by performing 150,000 objective function evaluations, and a stopping criterion of $1\times {10}^{-12}$ tolerance was employed for the SGO and the other metaheuristic algorithms. For statistical aims, 100 optimization runs are considered when calculating the mean and standard deviation of the optimization results. Furthermore, in dealing with the SGO and other alternatives, a fixed random state is considered to carry out a comparative examination under the same conditions. It is also noteworthy that the utilized tolerance in this paper is selected for 50 test functions while a lower tolerance such as 10^–30^ or 10^–50^ could be utilized for a smaller number of test functions.

The outcomes of the SGO algorithm and other 6 metaheuristics are given in Tables 3 and 4, which display the best values achieved in 100 optimization runs, along with the mean and standard deviation values for each of the 25 mathematical test functions. The analysis shows that the SGO algorithm generally outperforms the other metaheuristic algorithms.

**Table 3:** The best results of metaheuristic algorithms considering mathematical test functions.

No	Alternative metaheuristic algorithms						
	ABC	ACO	FA	GA	PSO	WOA	SGO
*F*_*1*_	2.09E+01	2.09E+01	2.06E+01	2.17E+00	2.35E+00	0.00E+00	0.00E+00
*F*_*2*_	2.10E+02	2.21E+02	5.04E+01	9.68E−01	3.35E−01	0.00E+00	0.00E+00
*F*_*3*_	1.30E+07	2.48E+08	4.77E−01	1.74E−04	2.86E−07	0.00E+00	0.00E+00
*F*_*4*_	3.77E+10	4.99E+10	1.78E+10	1.73E−02	0.00E+00	0.00E+00	0.00E+00
*F*_*5*_	1.03E+01	1.03E+01	6.10E−06	0.00E+00	6.36E−08	0.00E+00	0.00E+00
*F*_*6*_	− 5.28E−01	− 5.25E−01	− 9.66E−01	− 1.00E+00	− 1.00E+00	− 1.00E+00	− 1.00E+00
*F*_*7*_	1.63E+07	1.82E+07	2.99E+04	2.95E+01	6.67E−01	6.67E−01	9.99E−01
*F*_*8*_	0.00E+00	0.00E+00	0.00E+00	0.00E+00	0.00E+00	− 9.92E−01	0.00E+00
*F*_*9*_	− 7.82E−05	− 1.41E−05	− 9.96E−01	− 1.00E+00	− 1.00E+00	− 1.00E+00	− 1.00E+00
*F*_*10*_	4.61E+01	5.68E+01	3.44E+01	1.05E−02	3.02E−08	0.00E+00	0.00E+00
*F*_*11*_	4.51E+06	4.51E+06	6.06E+03	1.77E−05	0.00E+00	0.00E+00	0.00E+00
*F*_*12*_	1.18E+30	4.34E+31	2.58E+33	4.63E+23	2.08E+08	0.00E+00	0.00E+00
*F*_*13*_	− 6.98E+00	− 5.39E+00	− 1.54E+01	− 4.14E+01	− 4.97E+01	− 9.90E+01	− 9.90E+01
*F*_*14*_	6.79E+02	7.93E+02	3.54E+01	1.98E+01	4.36E+00	1.84E−02	6.45E−12
*F*_*15*_	2.00E+00	2.00E+00	2.10E+00	2.00E+00	2.00E+00	2.00E+00	2.00E+00
*F*_*16*_	4.60E+01	4.64E+01	2.79E+01	2.70E+01	2.89E+01	0.00E+00	0.00E+00
*F*_*17*_	1.42E+05	1.74E+05	4.22E+04	2.35E+04	1.15E+04	0.00E+00	0.00E+00
*F*_*18*_	2.70E+04	3.70E+04	1.18E+02	3.20E+00	1.87E−02	0.00E+00	0.00E+00
*F*_*19*_	4.51E−01	4.51E−01	3.73E−08	0.00E+00	0.00E+00	0.00E+00	0.00E+00
*F*_*20*_	1.44E+03	1.49E+03	5.22E+02	3.82E+01	1.10E+02	0.00E+00	0.00E+00
*F*_*21*_	6.57E+11	6.57E+11	6.57E+11	1.45E+03	2.03E−02	3.03E+03	7.93E+04
*F*_*22*_	8.85E+05	9.13E+05	6.13E+02	4.37E+01	2.82E+01	1.31E+01	2.30E−04
*F*_*23*_	8.47E+08	8.47E+08	3.61E+07	4.87E+02	8.57E+01	9.51E+01	2.40E−06
*F*_*24*_	4.54E+01	4.91E+01	3.57E+01	2.34E+00	1.70E+00	0.00E+00	0.00E+00
*F*_*25*_	1.05E+09	1.05E+09	5.38E+08	1.90E−03	0.00E+00	0.00E+00	0.00E+00

**Table 4:** The outcomes’ mean and standard deviation for metaheuristic algorithms considering mathematical test functions.

No	Data	Alternative metaheuristic algorithms						
		ABC	ACO	FA	GA	PSO	WOA	SGO
*F1*	*Mean*	2.11E+01	2.11E+01	2.07E+01	2.62E+00	3.86E+00	0.00E+00	0.00E+00
	*Std*	6.28E−02	5.82E−02	3.40E−02	2.12E−01	5.88E−01	0.00E+00	0.00E+00
*F2*	*Mean*	2.33E+02	2.49E+02	6.41E+01	5.40E+00	2.26E+00	0.00E+00	6.15E−08
	*Std*	8.76E+00	1.01E+01	5.93E+00	2.90E+00	1.36E+00	0.00E+00	6.15E−07
*F3*	*Mean*	3.70E+10	5.79E+12	6.80E−01	8.11E−04	5.76E−03	0.00E+00	0.00E+00
	*Std*	7.69E+10	1.46E+13	1.01E−01	4.48E−04	1.35E−02	0.00E+00	0.00E+00
*F4*	*Mean*	5.73E+10	7.20E+10	2.29E+10	3.53E−01	7.18E+02	0.00E+00	0.00E+00
	*Std*	7.96E+09	7.13E+09	2.03E+09	3.22E−01	3.84E+03	0.00E+00	0.00E+00
*F5*	*Mean*	1.67E+01	1.75E+01	3.20E−05	3.90E−14	1.13E−06	0.00E+00	0.00E+00
	*Std*	1.99E+00	2.02E+00	2.69E−05	3.90E−13	7.12E−07	0.00E+00	0.00E+00
*F6*	*Mean*	− 4.80E−01	− 4.79E−01	− 9.50E−01	− 9.44E−01	− 9.97E−01	− 8.22E−01	− 6.00E−01
	*Std*	1.04E−02	1.04E−02	6.90E−03	2.17E−02	4.61E−03	1.02E−01	2.26E−01
*F7*	*Mean*	2.60E+07	2.72E+07	5.59E+04	7.60E+01	2.06E+01	6.67E−01	1.00E+00
	*Std*	3.06E+06	3.05E+06	1.32E+04	3.63E+01	3.26E+01	1.84E−06	2.07E−04
*F8*	*Mean*	0.00E+00	0.00E+00	0.00E+00	0.00E+00	0.00E+00	− 9.58E−01	0.00E+00
	*Std*	0.00E+00	0.00E+00	0.00E+00	0.00E+00	0.00E+00	2.58E−02	0.00E+00
*F9*	*Mean*	− 6.23E−06	− 1.96E−06	− 9.94E−01	− 1.00E+00	− 1.00E+00	− 1.00E+00	− 1.00E+00
	*Std*	8.73E−06	1.94E−06	1.11E−03	1.18E−05	1.03E−03	0.00E+00	0.00E+00
*F10*	*Mean*	6.08E+01	6.80E+01	3.88E+01	3.35E−02	8.19E−02	5.93E−04	0.00E+00
	*Std*	4.38E+00	3.37E+00	1.69E+00	1.40E−02	1.44E−01	4.22E−03	0.00E+00
*F11*	*Mean*	6.52E+06	6.78E+06	1.37E+04	1.81E−04	0.00E+00	0.00E+00	0.00E+00
	*Std*	6.98E+05	7.54E+05	3.88E+03	1.46E−04	0.00E+00	0.00E+00	0.00E+00
*F12*	*Mean*	2.01E+30	7.09E+31	1.03E+34	5.27E+26	1.52E+20	0.00E+00	0.00E+00
	*Std*	4.45E+29	1.64E+31	2.80E+33	1.21E+27	1.46E+21	0.00E+00	0.00E+00
*F13*	*Mean*	− 5.40E+00	−3.68E+00	− 1.07E+01	− 3.53E+01	− 3.43E+01	− 9.87E+01	− 9.90E+01
	*Std*	5.05E−01	5.51E−01	1.50E+00	2.50E+00	5.38E+00	3.33E+00	0.00E+00
*F14*	*Mean*	8.44E+02	9.51E+02	5.79E+01	4.16E+01	1.50E+01	1.09E−01	4.88E+00
	*Std*	5.90E+01	6.19E+01	1.15E+01	1.32E+01	4.88E+00	1.49E−01	4.81E+00
*F15*	*Mean*	2.00E+00	2.00E+00	2.12E+00	2.00E+00	2.00E+00	2.00E+00	2.00E+00
	*Std*	0.00E+00	0.00E+00	6.66E−03	0.00E+00	3.79E−12	0.00E+00	0.00E+00
*F16*	*Mean*	4.68E+01	4.73E+01	3.03E+01	3.16E+01	3.33E+01	1.47E−02	3.62E−08
	*Std*	2.51E−01	2.52E−01	7.15E−01	1.28E+00	1.61E+00	1.01E−01	1.42E−07
*F17*	*Mean*	1.61E+05	2.01E+05	5.01E+04	3.87E+04	2.39E+04	0.00E+00	0.00E+00
	*Std*	7.99E+03	9.00E+03	2.91E+03	6.21E+03	5.00E+03	0.00E+00	0.00E+00
*F18*	*Mean*	4.97E+04	6.13E+04	2.06E+02	9.24E+00	4.80E−01	0.00E+00	0.00E+00
	*Std*	6.98E+03	8.93E+03	4.32E+01	2.50E+00	9.22E−01	0.00E+00	0.00E+00
*F19*	*Mean*	1.18E+00	1.30E+00	1.50E−07	1.11E−09	0.00E+00	0.00E+00	0.00E+00
	*Std*	3.11E−01	3.37E−01	8.03E−08	3.94E−09	0.00E+00	0.00E+00	0.00E+00
*F20*	*Mean*	1.57E+03	1.62E+03	6.11E+02	5.49E+01	1.78E+02	0.00E+00	0.00E+00
	*Std*	4.58E+01	4.78E+01	3.56E+01	5.48E+00	3.66E+01	0.00E+00	0.00E+00
*F21*	*Mean*	8.60E+11	8.87E+11	8.87E+11	4.17E+03	2.55E+04	1.34E+04	1.52E+05
	*Std*	6.21E+10	6.97E+10	6.97E+10	2.18E+03	1.47E+05	5.30E+03	6.45E+04
*F22*	*Mean*	1.14E+06	1.16E+06	1.01E+03	8.81E+01	6.74E+01	3.13E+01	2.36E+01
	*Std*	9.85E+04	1.04E+05	2.98E+02	1.86E+01	2.04E+01	1.44E+01	9.40E+01
*F23*	*Mean*	1.12E+09	1.14E+09	6.15E+07	8.83E+02	3.58E+02	9.57E+01	9.61E+01
	*Std*	8.38E+07	8.69E+07	1.12E+07	5.02E+02	6.16E+02	3.11E−01	1.38E+01
*F24*	*Mean*	4.98E+01	5.25E+01	3.96E+01	2.99E+00	3.33E+00	1.32E−01	0.00E+00
	*Std*	1.67E+00	1.30E+00	9.87E−01	2.59E−01	7.63E−01	6.17E−02	0.00E+00
*F25*	*Mean*	1.39E+09	1.42E+09	6.99E+08	1.81E−02	0.00E+00	0.00E+00	0.00E+00
	*Std*	9.79E+07	1.12E+08	5.84E+07	1.69E−02	0.00E+00	0.00E+00	0.00E+00

The mean of objective function evaluations for each function considering the 100 optimization runs performed by the SGO and alternative algorithms demonstrate that the SGO outperforms a fast optimization process in most cases, whereas there is no need for completing the predefined 150,000 objective function evaluations for achieving the tolerance of $1\times {10}^{-12}$. The mean of objective function evaluations for SGO is 43,163.86, while WOA with 66,629.20, PSO with 135,698.30, GA with 141,524.40, ACO with 144,009.60, ABC with 144,011.90 and FA with 150,000 have the second to seventh ranks.

**Figure 4:**
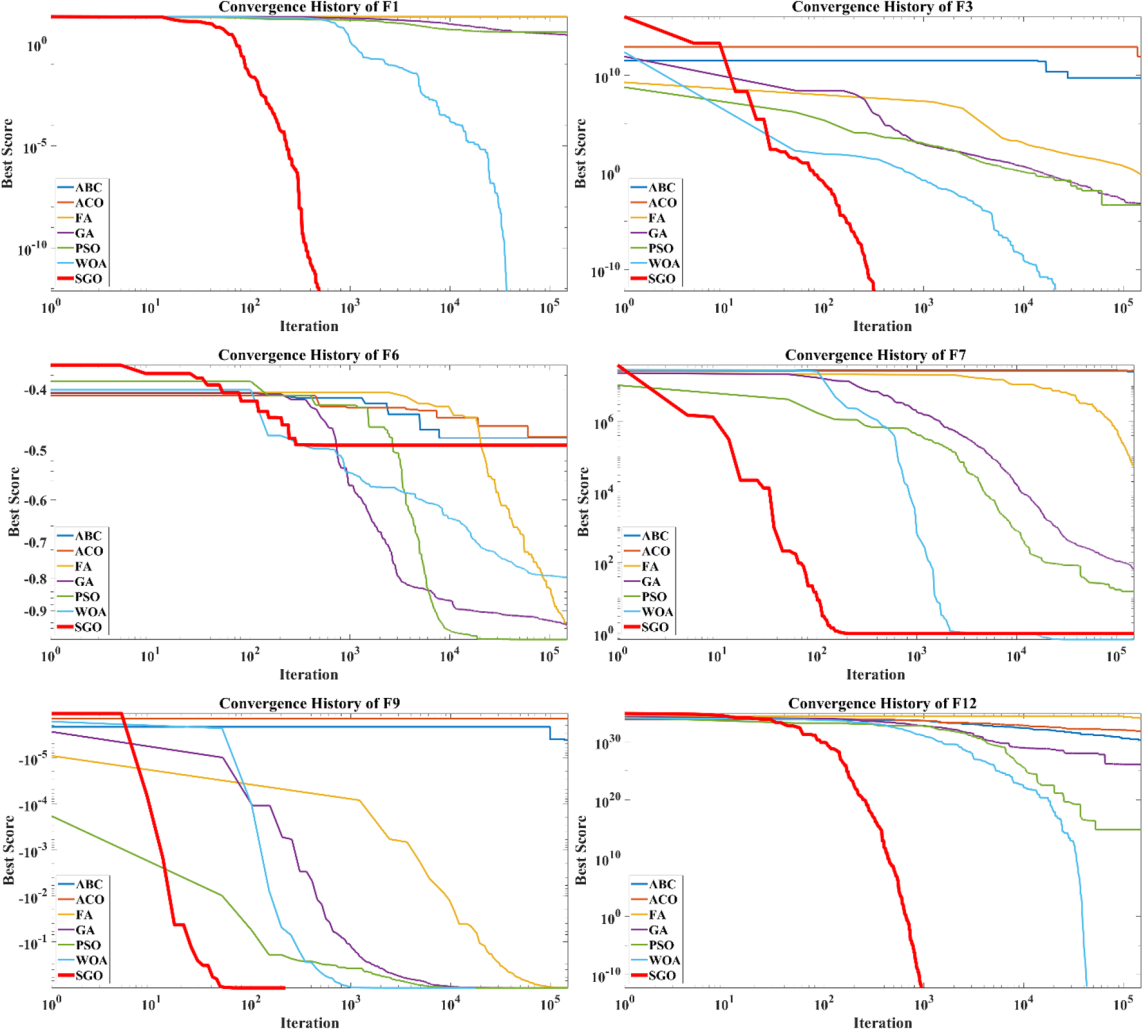

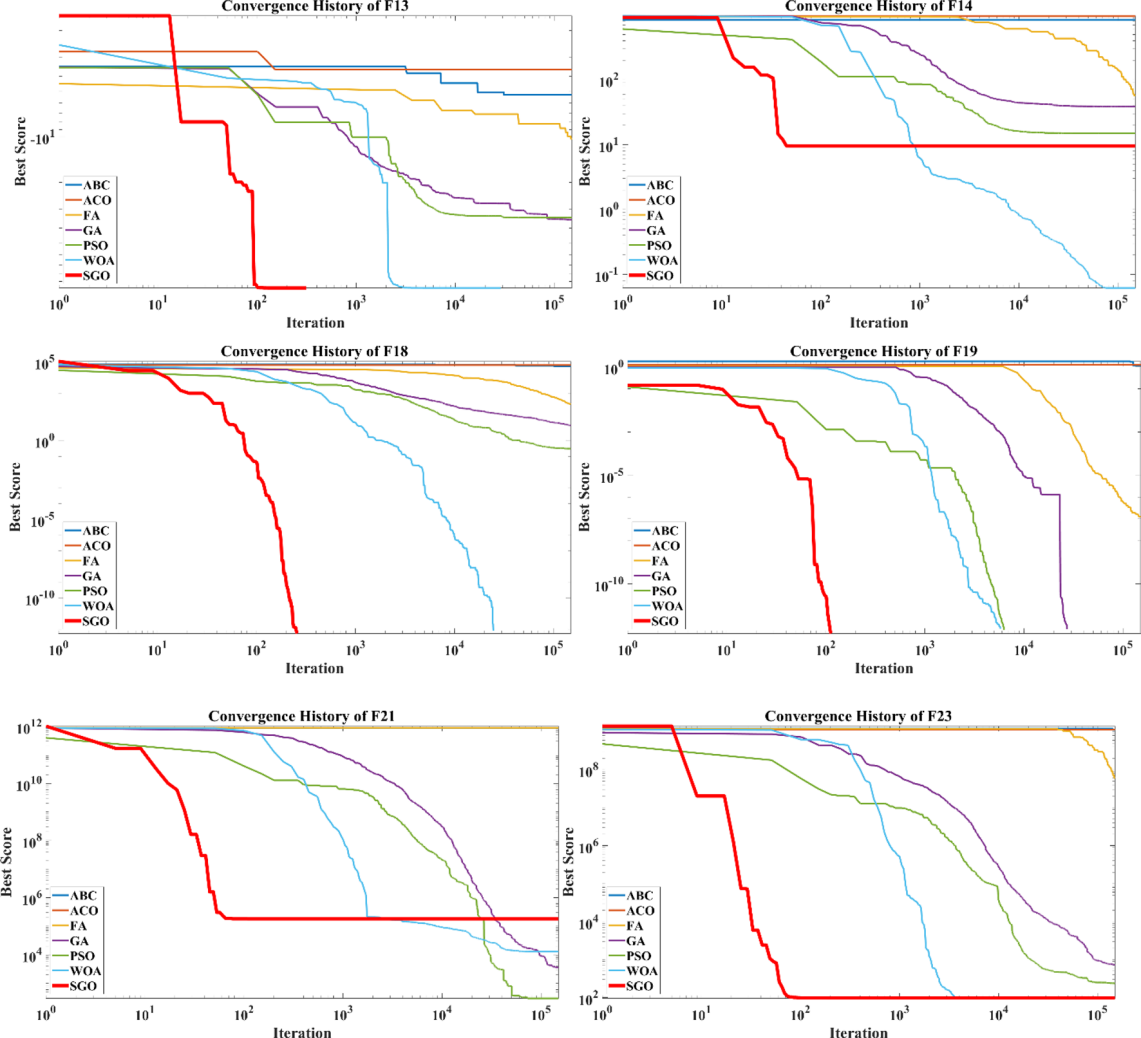
Convergence curves of metaheuristics regarding the median runs.

The convergence curves of different metaheuristic algorithms alongside the SGO are depicted in Fig. 2 in which the median run is used in each algorithm in dealing with each test function. It is obvious that the SGO has better convergence behavior alongside the WOA comparing to other methods. Based on the results, the SGO is capable of converging to global best minimum by considering the $1\times {10}^{-12}$ tolerance in a faster way in dealing with 21 test functions while for the other 4, the results of other algorithms are competitive. Based on the results of Table 4 and Fig. 2, the WOA shows better convergence behavior in dealing with F7 and F14. Regarding F21, the results of GA, PSO and WOA are better than the SGO while for F6, the results of GA, PSO, FA and WOA are more competitive than the results of SGO (Fig. 4).

## Statistical analysis

In addition to presenting the mean and standard deviation of the outcomes, the authors recognize the limitation of these measures in providing a comprehensive evaluation of algorithm performance when dealing with test functions. As such, a comprehensive statistical analysis has been conducted to address this issue, and the most relevant statistical tests have been utilized to evaluate the results. This approach provides a more in-depth analysis of the algorithms' capabilities and can yield more accurate and reliable conclusions regarding their performance. By conducting a comprehensive statistical analysis, the study aims to provide a more robust evaluation of the SGO algorithm and its performance compared to other metaheuristics. The paper employs various statistical tests to analyze and evaluate the performance of the miscellaneous metaheuristic algorithms used in dealing with the test functions. These tests include the Kolmogorov Smirnov (KS) test, which assesses the normality of results, the Mann Whitney (MW) test, which compares the ranks of different metaheuristics two by two, and the Kruskal Wallis (KW) test, which compares the mean ranks of the metaheuristic algorithms to determine the overall rankings. By using these statistical tests, the paper provides a more comprehensive evaluation of the algorithms, beyond just the mean and standard deviation of results.

### Kolmogorov Smirnov (KS) test

Table 5 shows the results of the Kolmogorov–Smirnov test used to identify whether to use non-parametric or parametric statistical tests for the collected data set, comprising the collection of mathematical test functions and other metaheuristic algorithms as alternatives. If the p-value of this test is less than 0.05, non-parametric tests are necessary, whereas if the p-value is higher than 0.05, parametric tests can be used. For future studies, the MW and KW tests, which are non-parametric statistical tests, should be selected based on the findings of the KS test.

**Table 5:** The KS test outcomes (p-values) of different metaheuristic algorithms.

Main algorithm	Data type	Alternative Metaheuristic Algorithms					
		ABC	ACO	FA	GA	PSO	WOA
SGO	Min	9.36E−09	9.36E−09	5.57E−08	1.52E−06	1.12E−04	9.90E−01
	Mean	1.52E−06	1.52E−06	1.52E−06	1.12E−04	3.97E−04	8.77E−01
	Std	6.95E−06	6.95E−06	6.95E−06	1.12E−04	3.97E−04	6.49E−01
	Fun. Evl	6.95E−06	6.95E−06	1.52E−06	6.95E−06	6.95E−06	2.92E−05

### Mann Whitney (MW) test

The MW test is conducted to compare the effectiveness of a wide variety of metaheuristics by considering the sum of their ranks. This test’s null hypothesis is that the two chosen variables from different data sets have the same statistical behavior. The results of the MW test, which are given in Table 6, show the sum of ranks for various metaheuristics in dealing with the test functions. The bold values in the table show that the metaheuristics with lower sum of ranks have better statistical behavior. The majority of the outcomes indicate that the proposed SGO algorithm has a lower sum of ranks, indicating its superiority over other algorithms. It is important to mention that the MW test can be a valuable tool for comparing the statistical performance of various algorithms, particularly in terms of their capacity to identify the global optimum.

**Table 6:** The MW test outcomes (summation of ranks) of different metaheuristic algorithms.

Main algorithm	Data type	Alternative metaheuristic algorithms					
		ABC	ACO	FA	GA	PSO	WOA
SGO	Min	914.50	914.50	914.50	861.50	822.50	646.00
		360.50	360.50	360.50	413.50	452.50	629.00
	Mean	895.50	895.50	883.50	832.50	815.50	652.00
		379.50	379.50	391.50	442.50	459.50	623.00
	Std	868.00	868.00	864.50	827.00	815.00	672.00
		407.00	407.00	410.50	448.00	460.00	603.00
	Fun. Evl	849.00	847.00	862.50	851.00	844.00	780.50
		426.00	428.00	412.50	424.00	431.00	494.50

### Kruskal Wallis (KW) test

The KW test is a statistical test that is frequently employed to compare the rankings of multiple variables across various datasets. In contrast to the MW and W tests, which are executed in a pairwise manner based on the summation and average of ranks, the KW test compares the average of ranks across datasets simultaneously. Lower mean ranks in a dataset are indicative of better statistical behavior in this test. Table 7 presents the statistical analysis of the performance of different metaheuristics on mathematical test functions using the KW test. The algorithms with the lowest mean ranks are bolded, highlighting their superior performance compared to other algorithms. The SGO algorithm consistently exhibited lower mean ranks in all cases, indicating its superiority over other algorithms.

**Table 7:** The KW test outcomes, including the mean of the ranks regarding mathematical test functions.

Rankings	Mathematical functions							
	Min		Mean		Std		Fun. Evl	
	Algorithms	Mean of ranks	Algorithms	Mean of ranks	Algorithms	Mean of ranks	Algorithms	Mean of ranks
1	**SGO**	**38.06**	**SGO**	**42.02**	**SGO**	**44.42**	**SGO**	**39.64**
2	WOA	42.40	WOA	43.88	WOA	49.72	WOA	52.92
3	PSO	76.80	PSO	84.04	GA	89.32	PSO	96.36
4	GA	87.84	GA	85.72	PSO	90.24	GA	102.20
5	FA	116.74	FA	111.80	FA	108.24	ACO	106.88
6	ABC	126.46	ABC	123.56	ABC	116.84	ABC	107.00
7	ACO	127.70	ACO	124.98	ACO	117.22	FA	111.00
*Chi-sq*	85.12		71.54		53.99		82.66	
*Prob* > *Chi-sq*	3.11E−16		1.98E−13		7.41E−10		1.01E−15	

Significant values are in bold.

## Computational complexity and cost analysis

Recent research has focused on developing algorithms that can optimize complex problems in a time- and computationally-efficient manner. Researchers have employed the computational complexity methods of the CEC 2020 benchmark suite on problems that have boundaries to overcome this challenge. In this suite, T_0_ measures the specific mathematical process’ run time, while T_1_ measures the computational time required for 200,000 function evaluations of the G1 function. T_2_ measures the mentioned algorithms’ computational time, including SGO in this study, for 200,000 function evaluations of the G_1_ function, and ${\widehat{\mathrm{T}}}_{2}$ is the mean value of T_2_ calculated five times^117^. Table 8 presents the outcomes of the computational time complexity of SGO and different algorithms using these procedures, demonstrating the superiority of the SGO algorithm regarding computational efficiency.

**Table 8:** Different algorithms’ computational time complexity concerning the CEC 2020 complexity process.

Metaheuristics	Properties	Results (s)
*IMODE*	*T0*	0.01117
	*T1*	0.2235
	${\widehat{T}}_{2}$	0.3330
	$({\widehat{T}}_{2}-T1)/T0$	0.9780
*j2020*	*T0*	0
	*T1*	0.0465
	${\widehat{T}}_{2}$	0.1818
	$({\widehat{T}}_{2}-T1)/T0$	Inf
*GSK*	*T0*	0.0411
	*T1*	1.12E−05
	${\widehat{T}}_{2}$	1.76E−05
	$({\widehat{T}}_{2}-T1)/T0$	1.58E−04
*SGO (Present Study)*	*T0*	0.0226
	*T1*	0.0142
	${\widehat{T}}_{2}$	3.9279
	$({\widehat{T}}_{2}-T1)/T0$	173.1725

The "Big O notation" is a well-established mathematical notation that has widespread application in the scientific and mathematical domains, including in computational complexity studies for metaheuristic algorithms. To evaluate and contrast the effectiveness of different algorithms, it is common practice to assess their memory usage and execution time. However, while it is easy to set numerical values for the complexity of an algorithm, analyzing runtime concerns is a more complex issue that requires careful consideration. To eliminate the influence of computer and hardware constraints, other complexity procedures should be utilized for determining algorithmic complexity. The "Big O notation" is a widely-used term in computer science to quantify the required memory and run time of algorithms for comparative analysis. To compute the computational complexity of the SGO algorithm, the number of initial solution candidates (NP) and the problem dimension (*D*) are first calculated. The initialization phase of SGO has a computational complexity of *O(NP* × *D)*, while the complexity of evaluating the objective function is *O(NP)* × *O(F(x))*, in which *F(x)* represents the objective function of the problem being considered. Each iteration in the main search loop of SGOA has a computational complexity equivalent to the number of iterations (*MxIter*). Updating the positions of each solution candidate in the search space during offensive and defensive player movements on the playground has a computational complexity of *O(MxIter* × *NP* × *D* × *3)*. Finally, the objective function evaluation within the main search loop of SGOA has a computational complexity of *O(MxIter* × *NP* × *D* × *3)* × *O(F(x))*.

## Real-world constrained optimization problems of CEC 2020

**Figure 5:**
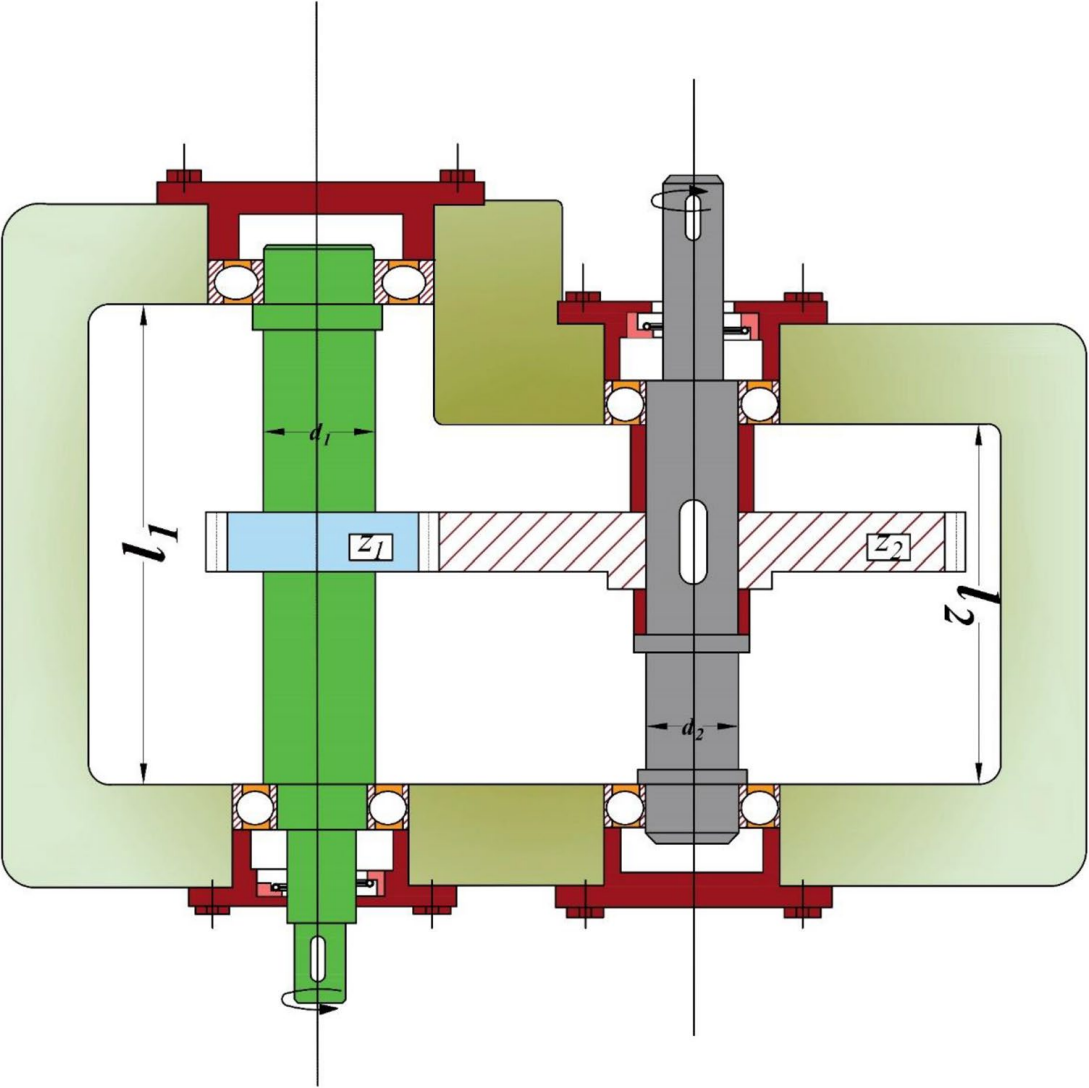
Schematic representation of the speed reducer problem.

**Figure 6:**
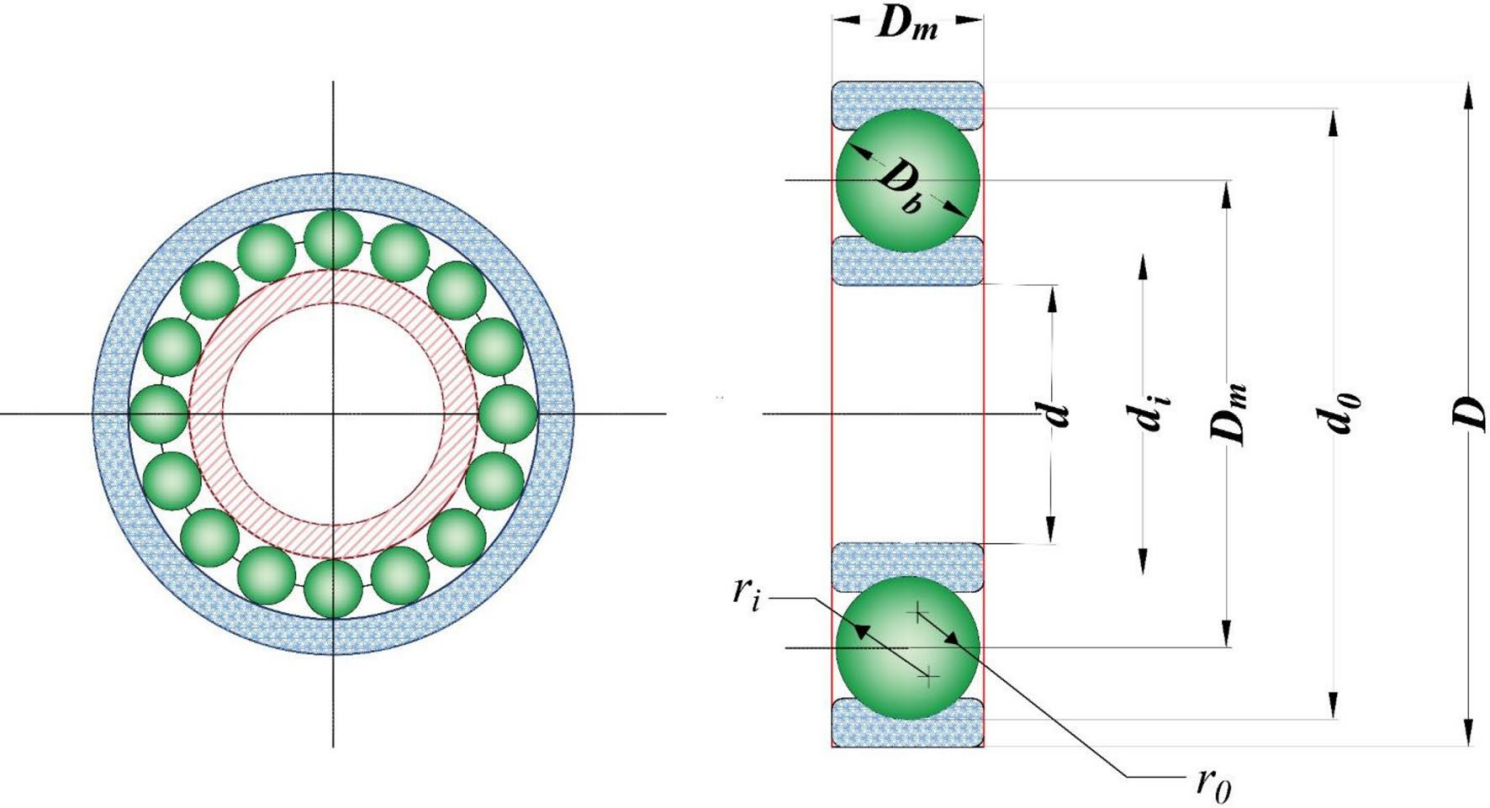
Schematic representation of the hydro-static thrust bearing.

**Figure 7:**
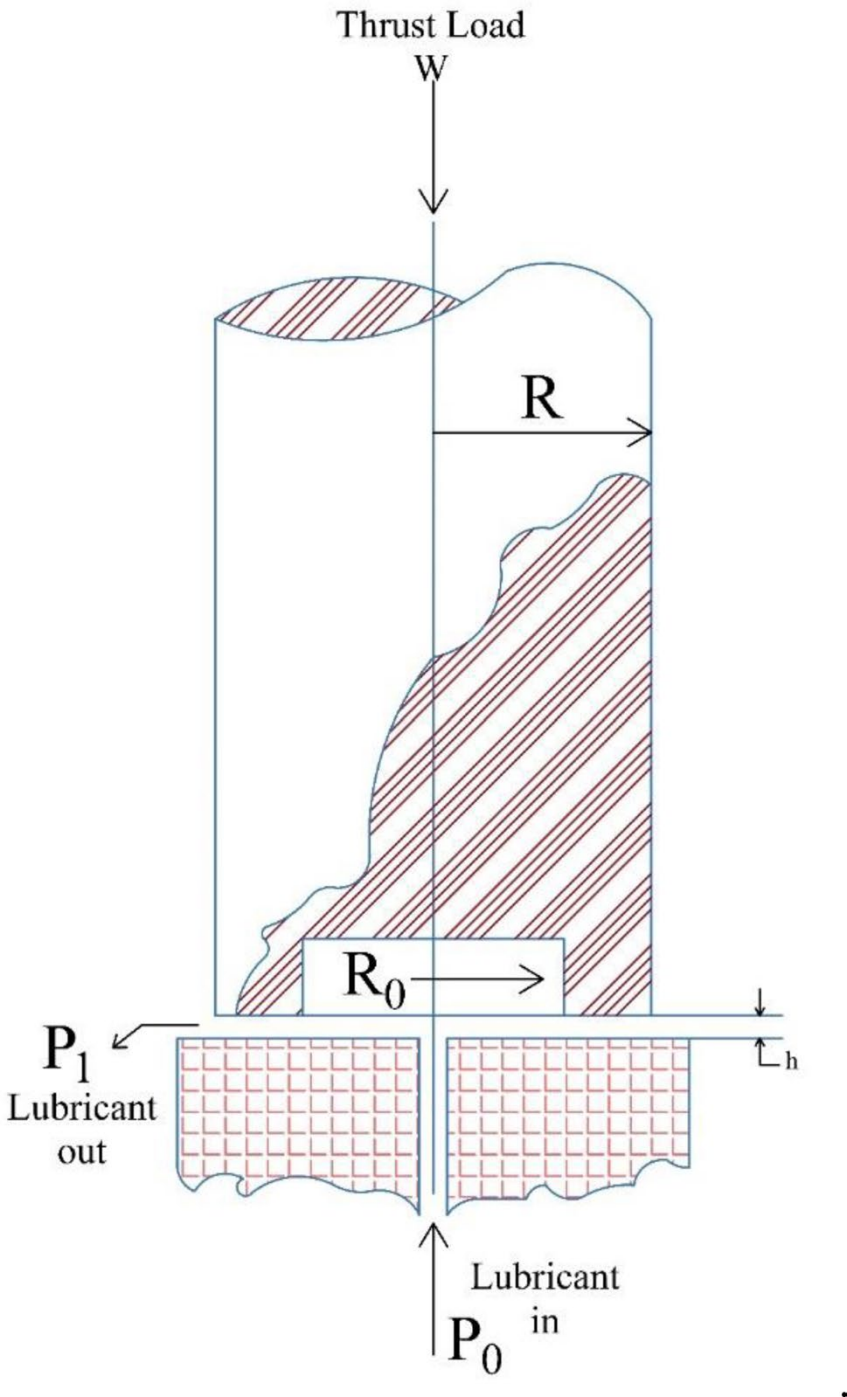
Schematic representation of the rolling element bearing problem.

In academic research, metaheuristic algorithms are typically assessed for their effectiveness in solving real-world optimization problems that involve both bound and design constraints. In this study, the CEC 2020 benchmark suite of real-world constraint optimization problems is utilized^55^, with Table 9 providing a summary of the engineering design problems and their mathematical formulations found in the literature. Figures 5, 6 and 7 present schematic representations of these problems. To ensure statistical validity, 30 independent optimization runs were carried out, each with 200,000 function evaluations. In order to handle the constraints of these problems, a prominent penalty technique with a static coefficient is employed in the current study.

**Table 9:** Real-world constrained optimization problems.

No. (CEC No.)	Name	D	g	h
H_1_ (RC15)	Speed reducer	7	11	0
H_2_ (RC25)	Hydro-static thrust bearing	4	7	0
H_3_ (RC28)	Rolling element bearing	10	9	0

*D* dimensions, *g* number of inequality constraints, *h* number of equality constraints.

The results of the speed reducer problem for SGO and other algorithms have been presented in Tables 10 and 11, which include optimal design variables and design constraints. The best optimization runs carried out by various techniques show that SGO can achieve a result of 2994.42, which is superior to other metaheuristics. In addition, the means and worst outcomes obtained by SGO are 2994.45 and 2994.48, respectively, which are more favourable than those of other methods. These statistical outcomes suggest that SGO is a highly effective and efficient algorithm for solving the speed reducer problem. The successful performance of SGO can be attributed to its capability to strike a balance between exploitation and exploration of the search space, as well as its efficient handling of the complex constraints inherent in this problem.

**Table 10:** Best outcomes of miscellaneous approaches for the speed reducer problem.

	ES^127^	SBS^128^	CSA^129^	DE^130^	MGA^35^	CGO^131^	Present study (SGO)
*Best*	*3025.0050000000*	*3008.0800000000*	*3000.9810000000*	*2994.4710660000*	*2994.4436490000*	*2994.4388690000*	**2994.4248154921**
*b*	*3.5061630000*	*3.5061220000*	*3.5015000000*	*3.5000000000*	*3.5000066840*	*3.5000079560*	3.5000001594
*m*	*0.7008310000*	*0.7000060000*	*0.7000000000*	*0.7000000000*	*0.7000000000*	*0.7000006560*	0.7000000038
*z*	*17.0000000000*	*17.0000000000*	*17.0000000000*	*17.0000000000*	*17.0000000000*	*17.0000008100*	17.0000000080
*l*_*1*_	*7.4601810000*	*7.5491260000*	*7.6050000000*	*7.3000000000*	*7.3000000000*	*7.3005419270*	7.3000048291
*l*_*2*_	*7.9621430000*	*7.8593300000*	*7.8181000000*	*7.7153199100*	*7.7153272500*	*7.7153576930*	7.7153232372
*d*_*1*_	*3.3629000000*	*3.3655760000*	*3.3520000000*	*3.3502146700*	*3.3505953620*	*3.3505423910*	3.3505412814
*d*_*2*_	*5.3090000000*	*5.2897730000*	*5.2875000000*	*5.2866544700*	*5.2866584470*	*5.2866579300*	5.2866545726
*g*_*1*_*(x)*	*− 0.0777000000*	*− 0.0755000000*	*− 0.0743000000*	* − 0.0739152*	*− 2.1550556750*	*− 2.1551222770*	*− *2.1550016575
*g*_*2*_*(x)*	*− 0.2013000000*	*− 0.1994000000*	*− 0.1983000000*	* − 0.1979985*	*− 98.1359464800*	*− 98.1371022200*	*− *98.1350284098
*g*_*3*_*(x)*	*− 0.4741000000*	*− 0.4562000000*	*− 0.4349000000*	* − 0.9999967*	*− 1.9253722120*	*− 1.9242737610*	*− *1.9251156798
*g*_*4*_*(x)*	*− 0.8971000000*	*− 0.8994000000*	*− 0.9008000000*	* − 0.9999995*	*− 18.3099259200*	*− 18.3096983400*	*− *18.3098982853
*g*_*5*_*(x)*	*− 0.0110000000*	*− 0.0132000000*	*− 0.0011000000*	*− 0.6668526000*	*− 0.0535900310*	*− 0.0004371520*	*− *0.0003184915
*g*_*6*_*(x)*	*− 0.0125000000*	*− 0.0017000000*	*− 0.0004000000*	* − 0.0000000*	*− 0.0019196020*	*− 0.0016664740*	*− *0.0000513607
*g*_*7*_*(x)*	*− 0.7022000000*	*− 0.7025000000*	*− 0.7025000000*	*− 0.7025000000*	*− 28.1000000000*	*− 28.0999882900*	*− *28.0999999300
*g*_*8*_*(x)*	*− 0.0006000000*	*− 0.0017000000*	*− 0.0004000000*	* − 0.0000000*	*− 0.0000095500*	*− 0.0000066800*	*− *0.0000002007
*g*_*9*_*(x)*	*− 0.5831000000*	*− 0.5826000000*	*− 0.5832000000*	* − 0.5833333*	*− 6.9999904520*	*− 6.9999933180*	*− *6.9999997993
*g*_*10*_*(x)*	*− 0.0691000000*	*− 0.0796000000*	*− 0.0890000000*	* − 0.0513257*	*− 0.3741069580*	*− 0.3747283410*	*− *0.3741929070
*g*_*11*_*(x)*	*− 0.0279000000*	*− 0.0179000000*	*− 0.0130000000*	* − 0.0000000*	*− 0.0000029600*	*− 0.0000340000*	*− *0.0000032074

Teeth module (m), face width (b), length of the first shaft between bearings (l1), the diameter of the first shaft (d1), number of teeth on pinion (z), length of the second shaft between bearings (l2), the diameter of the second shaft (d2).

Significant values are in bold and italics.

**Table 11:** Statistical results for the speed reducer problem considering different approaches.

Approaches	Best	Mean	Worst	Std-Dev
ES^127^	*3025.0050000000*	*3088.7778000000*	*3078.5918000000*	*NA*
SBS^128^	*3008.0800000000*	*3012.1200000000*	*3028.2800000000*	*NA*
CSA^129^	*3000.9810000000*	*3007.1997000000*	*3.0090000000*	*4.9634000000*
DE^130^	*2994.4710660000*	*2994.4710660000*	*2994.4710660000*	***0.0000000000***
MGA^35^	*2994.4388690000*	*2994.4706500000*	*2996.5582370000*	*4.72E−16*
CGO^131^	*2994.4436490000*	*2994.4653970000*	*2995.5049330000*	*0.1102820000*
Present Study (SGO)	**2994.4248154921**	**2994.4553460550**	**2994.4899883644**	0.0202512078

Significant values are in bold and italics.

Tables 12 and 13 present the best and statistical outcomes of various algorithms, including the proposed SGO, for the hydro-static thrust bearing design problem. The tables also display the optimal design variables and design constraints. The SGO algorithm performed the best among all the approaches by achieving 1618.95, while the best result from other approaches was obtained by CGO at 1621.24. Moreover, SGO's means and worst results were 1778 and 1910, respectively, which is superior to the results of other methods.

**Table 12:** Comparison of the best solutions for the hydro-static thrust bearing design problem.

	COM^132^	CGS^133^	EA^134^	TLBO^51^	MGA^35^	CGO^131^	Present Study (SGO)
*Best*	*2288.2268000000*	*2161.4215000000*	*1950.2860000000*	*1625.4427600000*	*1623.9809380000*	*1621.2461750000*	**1618.9878101725**
*R*	*7.1550000000*	*6.7780000000*	*6.2710000000*	*5.9557805026*	*5.9632415160*	*5.9634400230*	5.9560709112
*R*_*0*_	*6.6890000000*	*6.2340000000*	*12.9010000000*	*5.3890130519*	*5.3959079890*	*5.3955878610*	5.3889322344
*µ*	*0.0000083210*	*6.096 E−06*	*0.0000056050*	*0.0000053586*	*0.0000053800*	*0.0000053600*	0.0000053785
*Q*	*9.1680000000*	*3.8090000000*	*2.9380000000*	*2.2696559728*	*2.2822425050*	*2.2648221880*	2.2726159184
*g*_*1*_*(x)*	*− 11,086.7430000000*	*− 8329.7681000000*	*− 2126.8673400000*	*− 0.0001374735*	*− 144.9586796000*	*− 9.0788651780*	*− *3.3136822083
*g*_*2*_*(x)*	*− 402.4493000000*	*− 177.3527000000*	*− 68.0396000000*	*− 0.0000010103*	*− 1.1948020210*	*− 2.5136231960*	*− *0.1030173878
*g*_*3*_*(x)*	*− 35.0571960000*	*− 10.6845430000*	*− 3.7051910000*	*− 0.0000000210*	*− 0.3724500270*	*− 0.0021106440*	*− *0.3149154198
*g*_*4*_*(x)*	*− 0.0015420000*	*− 0.0006520000*	*− 0.0005590000*	*− 0.0003243625*	*− 0.0003291500*	*− 0.0003248340*	*− *0.0003268968
*g*_*5*_*(x)*	*− 0.4660000000*	*− 0.5440000000*	*− 0.6660000000*	*− 0.5667674507*	*− 0.5673335270*	*− 0.5678521610*	*− *0.5671386768
*g*_*6*_*(x)*	*− 0.0001440000*	*− 0.0007170000*	*− 0.0008050000*	*− 0.0009963614*	*− 0.0009963550*	*− 0.0009963660*	*− *0.0009963634
*g*_*7*_*(x)*	*− 563.6444010000*	*− 83.6182210000*	*− 849.7186830000*	*− 0.0000090762*	*− 4.1442588760*	*− 15.3591184600*	*− *3.2036645069

Bearing step radius (R), recess radius (R0), oil viscosity (µ), flow rate (Q).

Significant values are in bold and italics.

**Table 13:** Statistical results of different approaches for the hydro-static thrust bearing design problem.

Approaches	Best	Mean	Worst	Std-Dev
EGWO^135^	*1625.4646700000*	*1627.7441980000*	*1650.6987470000*	*3.8155469730*
JA^136^	*1625.4427100000*	*1796.8936700000*	*2104.3776000000*	*0.2100000000*
TLBO^138^	*1625.4427600000*	*1797.7079800000*	*2096.8012000000*	*0.1900000000*
MGA^35^	*1621.2461750000*	*1739.1567290000*	*1992.9613050000*	***0.1100000000***
CGO^131^	*1621.2461750000*	*1706.0414310000*	*1981.1732950000*	*64.4989571200*
Present Study (SGO)	**1618.9878101725**	**1778.2800314929**	**1910.7840110922**	83.0282071657

Significant values are in bold and italics.

Tables 14 and 15 present the statistical outcomes of the proposed SGO algorithm and other alternative algorithms in handling the maximization problem of rolling element bearing design, alongside the optimal design variables and constraints. The best optimization runs performed by various methods reveal that SGO achieves a TLBO result as 81,859.74, whereas the maximum value of the objective function is obtained by ALO with 85,546.63, taking into account that this particular problem is a maximization problem.

**Table 14:** Comparison of the best solutions for the rolling element bearing design problem.

	TLBO^137^	ABC^138^	GWO^138^	ALO^138^	MGA^35^	CGO^131^	Present Study (SGO)
*Best*	*81,859.7400000000*	*85,428.2495000000*	*85,529.0830000000*	***85,546.6377000000***	*83,912.8798300000*	*83,918.4925300000*	81,859.7402667138
*D*_*m*_	*21.4255900000*	*125.6599000000*	*125.7090000000*	*125.7180000000*	*125.0002787000*	*125.0000000000*	125.7190555843
*D*_*b*_	*125.7191000000*	*21.4086200000*	*21.4231600000*	*21.4252420000*	*21.8745119200*	*21.8750000000*	21.4255902292
*Z*	*11.0000000000*	*11.0000000000*	*11.0000000000*	*11.0000000000*	*10.7770658300*	*10.7770090500*	10.7258834098
*f*_*i*_	*0.5150000000*	*0.5150000000*	*0.5150000000*	*0.5150000000*	*0.5150008220*	*0.5150000000*	0.5150000007
*f*_*0*_	*0.5150000000*	*0.5150000000*	*0.5293220000*	*0.5157018000*	*0.5150029930*	*0.5150000000*	0.5150000000
*K*_*Dmin*_	*0.4242660000*	*0.4271660000*	*0.4208670000*	*0.4541646000*	*0.4059083530*	*0.4000000000*	0.4240900558
*K*_*Dmax*_	*0.6339480000*	*0.6688490000*	*0.6332960000*	*0.6464928000*	*0.6555880200*	*0.6462005260*	0.6974482305
*ε*	*0.3000000000*	*0.3000000000*	*0.3002240000*	*0.3000122000*	*0.3000041550*	*0.3000000000*	0.3000000000
*e*	*0.0688580000*	*0.0713860000*	*0.0200000000*	*0.0638003000*	*0.0775449260*	*0.0501524450*	0.0449615191
*ζ*	*0.7994980000*	*0.6000000000*	*0.6194320000*	*0.6107592000*	*0.6000000000*	*0.6000000000*	0.6071352958

Pitch diameter (Dm), ball diameter (Db), total number of balls (Z), inner raceway curvature coefficient (fi), and the outer raceway curvature coefficient (f0).

Significant values are in bold and italics.

**Table 15:** Statistical results of different approaches for the rolling element bearing design problem.

Approaches	Best	Mean	Worst	Std-Dev
TLBO^137^	*81,859.7400000000*	*81,438.9870000000*	*80,807.8551000000*	***0.6600000000***
ABC^138^	*85,428.2495000000*	***85,121.7544000000***	***83,859.0851000000***	*362.5700000000*
GWO^138^	*85,529.0830000000*	*83,395.0849000000*	43,543.4508000000	8224.5000000000
ALO^138^	***85,546.6377000000***	*84,032.8636000000*	*73,872.8164000000*	*3121.8000000000*
MGA^35^	*83,912.8798300000*	*83,892.2564700000*	*83,711.2131700000*	*23.6584100000*
CGO^131^	*83,918.4925300000*	*83,916.5974900000*	*83,829.8000000000*	*10.5358000000*
Present Study (SGO)	81,859.7402667138	80,404.8554383897	79,156.2618531067	719.1899843849

Significant values are in bold and italics.

## Discussion

Based on the provided results in the previous section, the capability of the proposed SGO algorithm have been investigated through different optimization problems. From mathematical functions point of view, the SGO can provide better competitive results including the best, mean, works and standard deviation while the SGO can provide lower mean of objective function evaluations. For 20 of the considered 25 mathematical test function, the SGO is capable of reaching to the global optimum point while the second best algorithm (WOA) is capable of reaching to global optima of 18 test functions. Regarding statistical test, the SGO can calculate lower mean and summation of ranks in competing with other alternative algorithms while the results of the WOA is also very close to the SGO’s outcomes. The minimum difference between the SGO and other approaches regarding MW test is for minimum values in which the summation of ranks for SGO and WOA are 646 and 629 respectively. Regarding the mean of ranks of different approaches, the maximum difference is between SGO and ACO for minimum values with mean ranks of 38.06 and 127.70 respectively. For the considered engineering optimization problem, the SGO can reach better optimum design in two of them by providing better statistical results too while for one of them, the SGO provides very competitive results. SGO can reach 2994.42 for the speed reducer problem which is the best among other approaches while the DE algorithm can calculate better standard deviation for this case. SGO is capable of calculating 1618.98 for the hydro-static thrust bearing design problem as the best among other methods while the MGA with 0.11 as standard deviation has better outcome than other methods.

## Conclusion and future directions

The Squid Game Optimizer (SGO) algorithm is a novel proposed metaheuristic algorithm inspired by the primary rules of a traditional Korean game. Metaheuristic algorithms are powerful optimization techniques that are widely used to solve complex optimization problems with various constraints and objectives. The SGO algorithm is a population-based optimization algorithm that mimics the behaviours of the players in the Squid Game by dividing the population into different groups based on their fitness values and applying different search strategies to each group. This methodology enables the SGO algorithm to effectively explore the search space and prevent being trapped in local optima. To assess the performance of the SGO algorithm, 25 unconstrained mathematical test functions were employed in this study. To ensure the statistical significance of the results, 100 independent optimization runs were executed. Additionally, the SGO algorithm was evaluated on two of the most recent CEC, specifically the CEC 2020 for bound constraint optimization and the CEC 2020 for real-world optimization. The CEC benchmarks are considered a widely recognized standard evaluation platform for optimization algorithms, which ensures a fair and unbiased comparison among different algorithms. The experimental results indicate that the SGO algorithm is highly competitive and exhibits superior performance compared to other well-known metaheuristics on a diverse set of optimization problems. The SGO algorithm can discover high-quality solutions with faster convergence rates, better solution accuracy, and higher diversity in the solution space. The SGO algorithm is also shown to be robust and scalable, and can effectively handle optimization problems with various degrees of complexity and dimensions. SGO has shown superior performance compared to other metaheuristic algorithms in dealing with unconstrained mathematical test functions and can converge to the global best solutions in most situations. Here are the main outcomes and key findings of the research:

- SGO was observed to have high computational efficiency, requiring the lowest number of objective function evaluations to reach the global optimum solution. This suggests that SGO has a promising potential to be used in various optimization problems that require efficient algorithms.
- By comparing various algorithms in a two-by-two way, the SGO could offer superior outcomes with smaller means of rankings in most cases based on the MW statistical test findings.
- KW statistical test’s outcomes, which includes the mean rank, suggest that in all evaluated datasets, the SGO algorithm outperforms other algorithms.
- The SGO algorithm was able to produce superior solutions than other approaches in the literature for the CEC 2020 on real-world problems’ constrained design examples.
- The optimal solution obtained by SGO in the speed reducer problem, based on the best optimization runs among various algorithms, is 2994.4248, which outperforms the results of other methods.
- SGO outperforms other algorithms in solving the hydro-static thrust bearing design problem, achieving the best result of 1618.9878. The closest result from other algorithms is for CGO, which achieved 1621.24.
- Considering the rolling element bearing design problem, SGO could provide 81,859.74, while the TLBO provides the best maximum value of the objective function in this case.

The experimental results clearly demonstrate that the SGO algorithm outperforms other well-established metaheuristic algorithms in terms of three critical factors: parameter-free, quick convergence, and the lowest possible objective function evaluation. The parameter-free nature of the SGO algorithm makes it more user-friendly and easier to implement in practical applications, eliminating the need for fine-tuning of parameters to achieve optimal performance. The quick convergence behaviour of the SGO algorithm enables it to discover high-quality solutions more efficiently, thereby reducing the overall computational time and cost of optimization. Finally, the lowest possible objective function evaluation of the SGO algorithm results in faster and more accurate optimization, making it a highly efficient and effective optimization technique. These three advantages of the SGO algorithm make it a promising tool for solving complex optimization problems in various fields, including real-size engineering design problems. The robustness and scalability of the SGO algorithm, as shown in the experimental results, suggest that it can effectively handle optimization problems with various degrees of complexity and dimensions, making it suitable for real-world applications.

The findings of this study have significant implications for future research in the field of evolutionary computation and optimization. The proposed SGO algorithm provides a novel and effective approach to solving complex optimization problems, and its three main advantages over other metaheuristic algorithms open up new avenues for research on optimization techniques with similar features. Moreover, the proposed SGO algorithm can be extended and customized for different applications by incorporating specific problem constraints and objectives, providing a highly adaptable optimization framework. Therefore, future studies should test the SGO algorithm on a wider range of optimization problems and further explore its potential in various real-world applications.

## Supplementary Information

Supplementary Information 1.
