# Supplementary figures and images for: Squid Game Optimizer (SGO): a novel metaheuristic algorithm
Source: Sci Rep. 2023 Apr 1;13:5373. doi: 10.1038/s41598-023-32465-z (PMC10066950; doi:10.1038/s41598-023-32465-z)

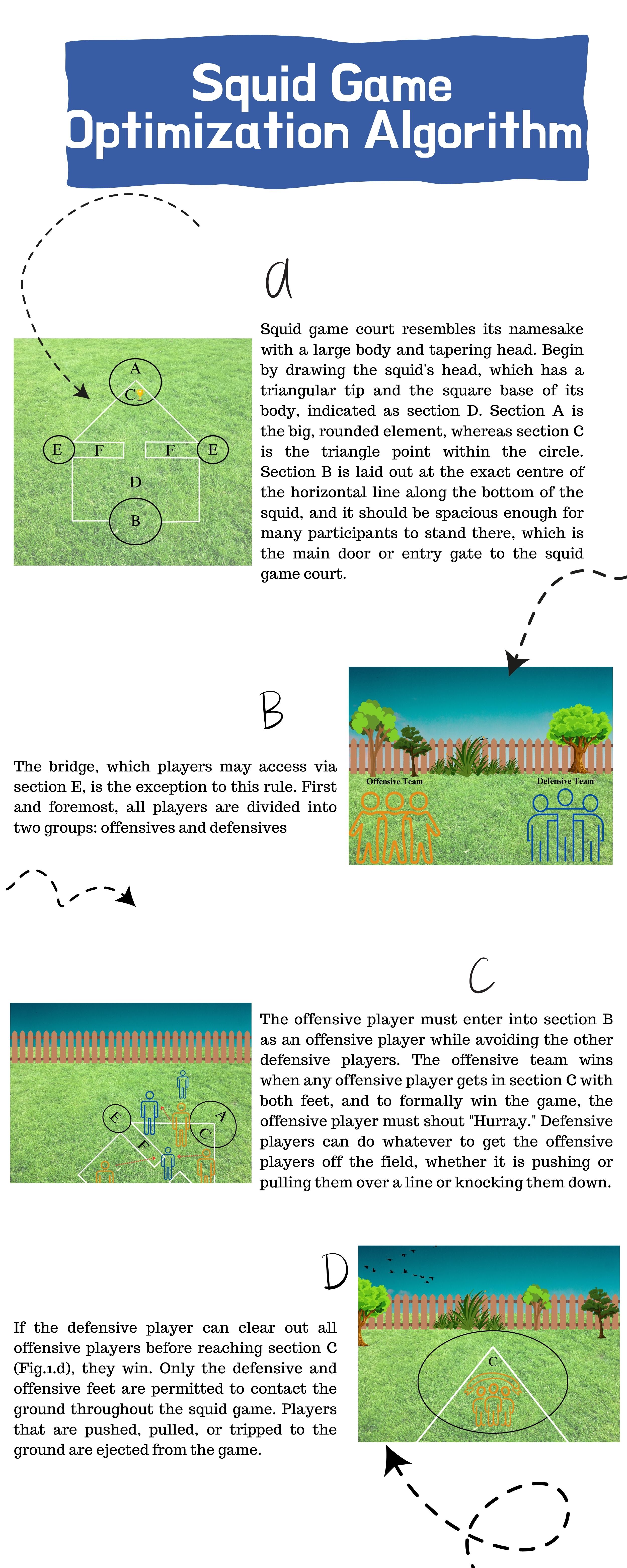

Supplement: Supplementary file 1 — Supplementary Information 1. [file 41598_2023_32465_MOESM1_ESM.jpg]
